# New insights in uranium bioremediation by cytochromes of the bacterium *Geotalea uraniireducens*

**DOI:** 10.1016/j.jbc.2024.108090

**Published:** 2024-12-14

**Authors:** Alexandre Almeida, David L. Turner, Marta A. Silva, Carlos A. Salgueiro

**Affiliations:** 1 Associate Laboratory i4HB – Institute for Health and Bioeconomy, NOVA School of Science and Technology, Universidade NOVA de Lisboa, Caparica, Portugal; 2 UCIBIO – Applied Molecular Biosciences Unit, Chemistry Department, NOVA School of Science and Technology, Universidade NOVA de Lisboa, Caparica, Portugal; 3 Instituto de Tecnologia Química e Biológica António Xavier, Universidade Nova de Lisboa, Oeiras, Portugal

**Keywords:** Electrogenic bacteria, electron transfer, multiheme *c*-type cytochromes, redox characterization, nuclear magnetic resonance (NMR)

## Abstract

The bacterium *Geotalea uraniireducens*, commonly found in uranium-contaminated environments, plays a key role in bioremediation strategies by converting the soluble hexavalent form of uranium (U(VI)) into less soluble forms (*e.g.*, U(IV)). While most of the reduction and concomitant precipitation of uranium occur outside the cells, there have been reports of important reduction processes taking place in the periplasm. In any case, the triheme periplasmic cytochromes are key players, either by ensuring an effective interface between the cell’s interior and exterior or by directly participating in the reduction of the metal. Therefore, understanding the functional mechanism of the highly abundant triheme cytochromes in *G. uraniireducens*’ is crucial for elucidating the respiratory pathways in this bacterium. In this work, a detailed functional characterization of the triheme cytochromes PpcA and PpcB from *G. uraniireducens* was conducted using NMR and visible spectroscopy techniques. Despite sharing high amino acid sequence identity and structural homology with their counterparts from *Geobacter sulfurreducens*, the results showed that the heme reduction potential values are less negative, the order of oxidation of the hemes is distinct, and the redox and redox-Bohr network of interactions revealed unprecedented functional mechanisms in the cytochromes of *G. uraniireducens*. In these cytochromes, the reduction potential values of the three heme groups are much more similar, resulting in a narrower range of values, that facilitates directional electron flow from the inner membrane, thereby optimizing the uranium reduction.

Within the complex diversity of respiratory processes naturally occurring in the microbial world, certain bacteria have gained recognition for their remarkable ability to overcome the limitations of the more standard ones. This is the case for the *Geobacter* bacteria, previously considered part of the *Proteobacteria* phylum and *Deltaproteobacteria* class. In fact, a recent phylogenetic analysis pinpointed incongruences in the trees for *Deltaproteobacteria* and a new reclassification was proposed (1). Consequently, the *Geobacter* bacteria are now part of the *Desulfobacterota* phylum, *Desulfuromonadia* class, *Geobacterales* order, and *Geobacteraceae* family, which also includes the genera *Geotalea* and *Citrifermentans* (for review, see (1)).

The bacteria from the *Geobacteraceae* family have emerged as crucial contributors to the dynamics of environmental microbiology due to their highly versatile respiratory pathways, which enable them to utilize both soluble and insoluble electron acceptors. A notable example of this versatility is provided by the use of distinct insoluble metal oxides as terminal electron acceptors, including, but not limited to, Fe(III), Cr(VI), U(VI), and Mn(IV) oxides (2, 3). Some of these metals, such as the hexavalent Cr(VI) and U(VI), are particularly concerning due to their carcinogenic and/or radioactive properties (4). Therefore, the reduction of such metals to less soluble forms and their subsequent precipitation have triggered the development of environmental bioremediation strategies (5, 6, 7). Additionally, these bacteria are well-known for their high level of current production in microbial fuel cells (MFC) in which electrode surfaces act as terminal electron acceptors. Consequently, they have also become attractive targets for applications in the bioenergy field (8, 9).

Within the *Geobacteraceae* family, significant progress has been made in elucidating the mechanisms that enable the use of extracellular electron acceptors. These mechanisms have been particularly clarified in *Geobacter sulfurreducens*, the first bacterium of the family to have its genome sequenced (10) and for which a genetic system was developed (11). Since then, twelve additional genomes from this bacterial family have been sequenced (10, 12, 13, 14, 15, 16, 17, 18). Their analysis has highlighted a high number of *c*-type cytochromes as a distinctive feature. The total number of cytochromes found in the periplasmic space, as well as, those associated with the inner and outer membranes varies among the different species. For instance, *G. sulfurreducens* contains at least 128 *c*-type cytochromes (19, 20), while *Geobacter uraniireducens*, now reclassified as *Geotalea uraniireducens*, has 105.

Originally obtained from uranium-contaminated subsurface sediments in Rifle, Colorado, *G. uraniireducens* has emerged as the prevalent organism during *in situ* uranium bioremediation (21). Contrarily to *G. sulfurreducens*, which requires direct contact to reduce Fe(III) oxides, encapsulated *G. uraniireducens* cells within microporous beads were able to reduce them, suggesting the export of soluble electron shuttles (22).

In the last decades, several studies have supported the high diversity of extracellular electron transfer mechanisms within the *Geobacteraceae* family members (17, 22, 23, 24). One striking example is the lack of correspondence between the effectiveness of current generation and Fe(III) oxide reduction rates by the different members (23). Despite the diversity of the extracellular electron transfer mechanisms employed, the multiheme cytochromes that hold the foundations of each mechanism are globally conserved. This includes the quinol oxidases associated with the inner membrane (CbcL or ImcH), outer membrane porin proteins (OmcB-based or Ext-cluster), and a family of periplasmic triheme cytochromes, with approximately 10 kDa and 70 residues each (25). The last group of cytochromes are the most abundant electron transfer proteins within the *Geobacteraceae* family (17, 26, 27, 28), and, besides their role in the electron transfer flow from the inner to the outer membrane electron transfer components (29, 30), they also function as strategic cellular capacitors and provide protection against oxidative stress (31, 32). Notably, while these cytochromes are highly conserved and homologous, they exhibit distinct functional properties in each bacterium (33, 34). Thus, given the environmental importance of *G. uraniireducens* and the lack of data on its electron transfer components, this study focuses on the periplasmic family of triheme cytochromes. The genome of *G. sulfurreducens* encodes five of these cytochromes, designated as PpcA-E (25, 35). Instead, four periplasmic homologs were identified for *G. uraniireducens* with gene annotation numbers *Gura_1303*, *Gura_3843*, *Gura_4121*, and *Gura_4124* (17). When compared to the amino acid sequences of their homologs from *G. sulfurreducens*, the triheme cytochromes from *G. uraniireducens* can be classified as PpcA (Gura_4121) and PpcB (Gura_3843) (Fig. 1 and Table 1). However, the other two cytochromes show low amino acid sequence identity with any of the remaining family members and are designated as PpcG (Gura_4124) and PpcH (Gura_1303).

**Figure 1:**
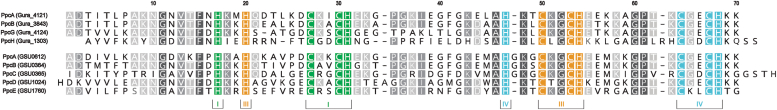
**Alignment of the amino acid sequences of the triheme periplasmic cytochromes from *Geotalea uraniireducens* with their homologs from *Geobacter sulfurreducens*.** Sequences were obtained from a BLAST analysis and aligned using Clustal Omega. Each cytochrome is identified with its NCBI access number. The residue numbers are indicated relative to cytochrome PpcA from *G. uraniireducens*. The heme-binding motifs (CXXCH) are highlighted in *green*, *orange*, and *blue* for hemes I, III, and IV, respectively. The hemes are numbered I, III, and IV, following the designation derived from their superimposition with those of the structurally homologous tetraheme cytochromes *c*_3_ (73). The same color code is used for the sixth axial ligand (histidine) of each heme. Residues that do not bind to the hemes but are fully conserved are colored in *dark gray*, whereas those with sequence identity in the range 100%–80% and 80%–60% are colored in *gray* and *light gray*, respectively. Residues showing <60% sequence similarity are left uncolored.

**Table 1:** Amino acid sequence identity (%) between the PpcA families from *Geotalea uraniireducens* (*Gu*) and *Geobacter sulfurreducens* (*Gs*)

Cytochrome	PpcA*Gs*	PpcB*Gs*	PpcC*Gs*	PpcD*Gs*	PpcE*Gs*
PpcA*Gu*	76	75	62	62	63
PpcB*Gu*	73	73	61	65	65
PpcG*Gu*	62	65	46	62	58
PpcH*Gu*	42	41	41	41	40

The percentage of identity was obtained from the Basic Local Alignment Search Tool (BLAST) (76).

Biochemical and biophysical studies on small multiheme *c*-type cytochromes with a low residue-to-heme ratio (approximately 20), such as the periplasmic triheme cytochromes from the *Geobacteraceae* family, have revealed significant differences in their functional and mechanistic properties, even among the most homologous proteins (36). In fact, a single amino acid replacement can lead to considerable changes in the the redox working range and/or the redox-Bohr properties and consequently in the functional mechanistic properties of the proteins (37). Therefore, to better understand and rationalize any eventual modification, this work provides a detailed biochemical and biophysical characterization of PpcA and PpcB from *G. uraniireducens* (hereafter referred to as PpcA*Gu* and PpcB*Gu*), within the context of existing data on the highly homologous cytochromes from *G. sulfurreducens*. Both cytochromes were heterologously expressed in *Escherichia coli*, and the combined use of UV-visible and NMR spectroscopic techniques allowed the characterization of the heme core architecture and global fold of the proteins. The study was further extended to the determination of the reduction potential values of each heme group and the entire network of intramolecular redox interactions. The results obtained were rationalized in the light of the data available for their homologs from *G. sulfurreducens* and to the typical environmental niche of *G. uraniireducens*.

## Results and discussion

### Production of cytochromes *PpcA*Gu and *PpcB*Gu

The cytochromes PpcA*Gu* and PpcB*Gu* are composed of 70 amino acids and have an isoelectric point of approximately 9 (determined by the pI/Mw tool program on the ExPASy Server - http://web.exp-asy.org/compute_pi/). The high isoelectric point is comparable to those of their homologs from *G. sulfurreducens*. The purification of PpcA*Gu* and PpcB*Gu* was carried out as previously described, involving ionic exchange followed by a final polishing step with size-exclusion chromatography. These two steps were adequate to obtain a homogeneous and pure sample, as observed in the size-exclusion chromatography elution profile and further confirmed by the SDS-PAGE (Fig. S2) and mass spectrometry. The determined molecular masses of pure PpcA*Gu* and PpcB*Gu* were 9.55 and 9.42 kDa, respectively, which agreed with the expected values (7.69 and 7.57 kDa for the PpcA*Gu* and PpcB*Gu* apo-proteins, respectively, plus 1.85 kDa for the three heme groups). The presence of the three heme groups was further confirmed by the pyridine hemochromogen assay. The molar extinction coefficients of both cytochromes were determined by the Lowry colorimetric assay at 552 nm (PpcA*Gu*) and 551 nm (PpcB*Gu*) in the reduced form. The values obtained (ε_552nm_ = 96 mM^−1^cm^−1^ and ε_551nm_ = 97 mM^−1^cm^−1^ for PpcA*Gu* and PpcB*Gu*, respectively) were used to calculate the purified protein yields for both proteins (3 mg per liter of culture medium).

### The heme groups of *PpcA*Gu and *PpcB*Gu are low-spin and axially coordinated by two histidine residues

**Figure 2:**
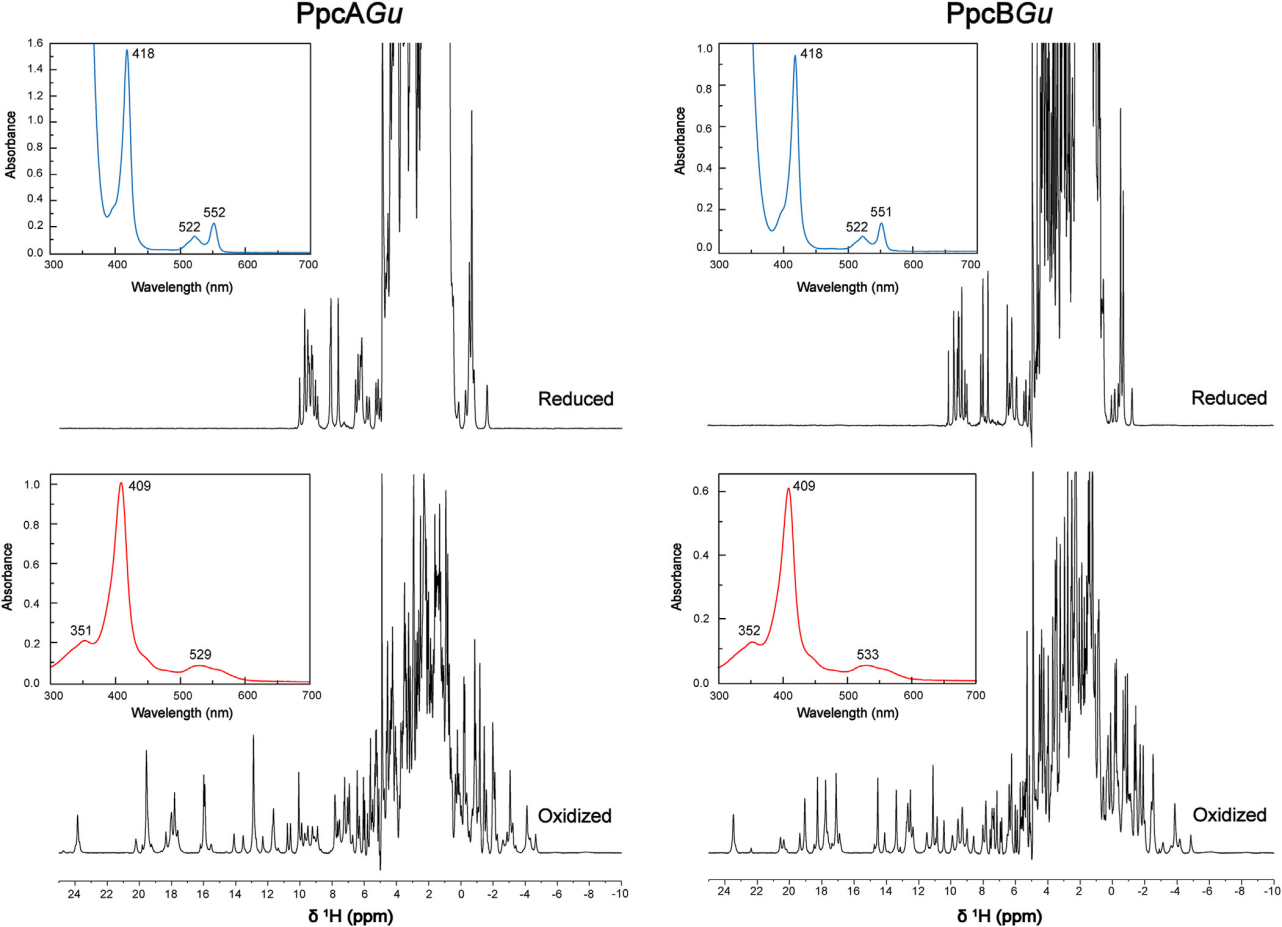
**1D**^**1**^**H-NMR and UV-visible spectral features of cytochromes PpcA*Gu* and PpcB*Gu* in the reduced (*upper panels*) and oxidized (*bottom panels*) states.** The absorption maxima of the UV-visible spectra of each cytochrome in both oxidized (*red line*) and reduced (*blue line*) states are labeled. The NMR spectra were acquired for 500 μM protein samples prepared in 80 mM sodium phosphate buffer with NaCl (final ionic strength of 250 mM), at pH 8, 15 °C. The NMR signals cover distinct spectral regions in the diamagnetic reduced state (−2 to 10.5 ppm) and in the paramagnetic oxidized state (−10 to −25 ppm).

The cytochromes PpcA*Gu* and PpcB*Gu* share a very high degree of sequence identity with their homologs from *G. sulfurreducens* (76% and 73%, respectively - see Table 1). The amino acid sequence alignment shows that the three heme-binding motifs containing the heme proximal axial ligands are conserved (His^31^, His^54^, and His^68^ for hemes I, III, and IV, respectively) as well as the distal ligands (His^17^, His^20^, and His^47^ for hemes I, III, and IV, respectively - Fig. 1). Therefore, all heme groups are expected to be low-spin and hexacoordinated. This was experimentally confirmed by UV-visible and NMR spectroscopic techniques obtained for the two cytochromes in both reduced and oxidized states (Fig. 2). The maxima of the UV-visible spectra (see insets in Fig. 2) are characteristics of low-spin hexacoordinated hemes (38), a feature independently confirmed by the acquisition of NMR spectra. In fact, these spectra are well-resolved, showing narrow signals in both the reduced and oxidized states (Fig. 2). In the reduced state, the signals are observed within a spectral range from −2 to 10.5 ppm. On the other hand, in the oxidized state, due to the unpaired electron of each heme, the NMR signals are more dispersed but still cover smaller spectral regions (−10 to 25 ppm) compared to those of high-spin hemes, which display very broad signals with chemical shifts above 40 ppm. Therefore, taking together the UV-visible and NMR data, it can be concluded that each heme is diamagnetic when in the reduced state (S = 0) and paramagnetic (S = 1/2) in the oxidized state.

### Structural insights of cytochromes *PpcA*Gu and *PpcB*Gu

**Figure 3:**
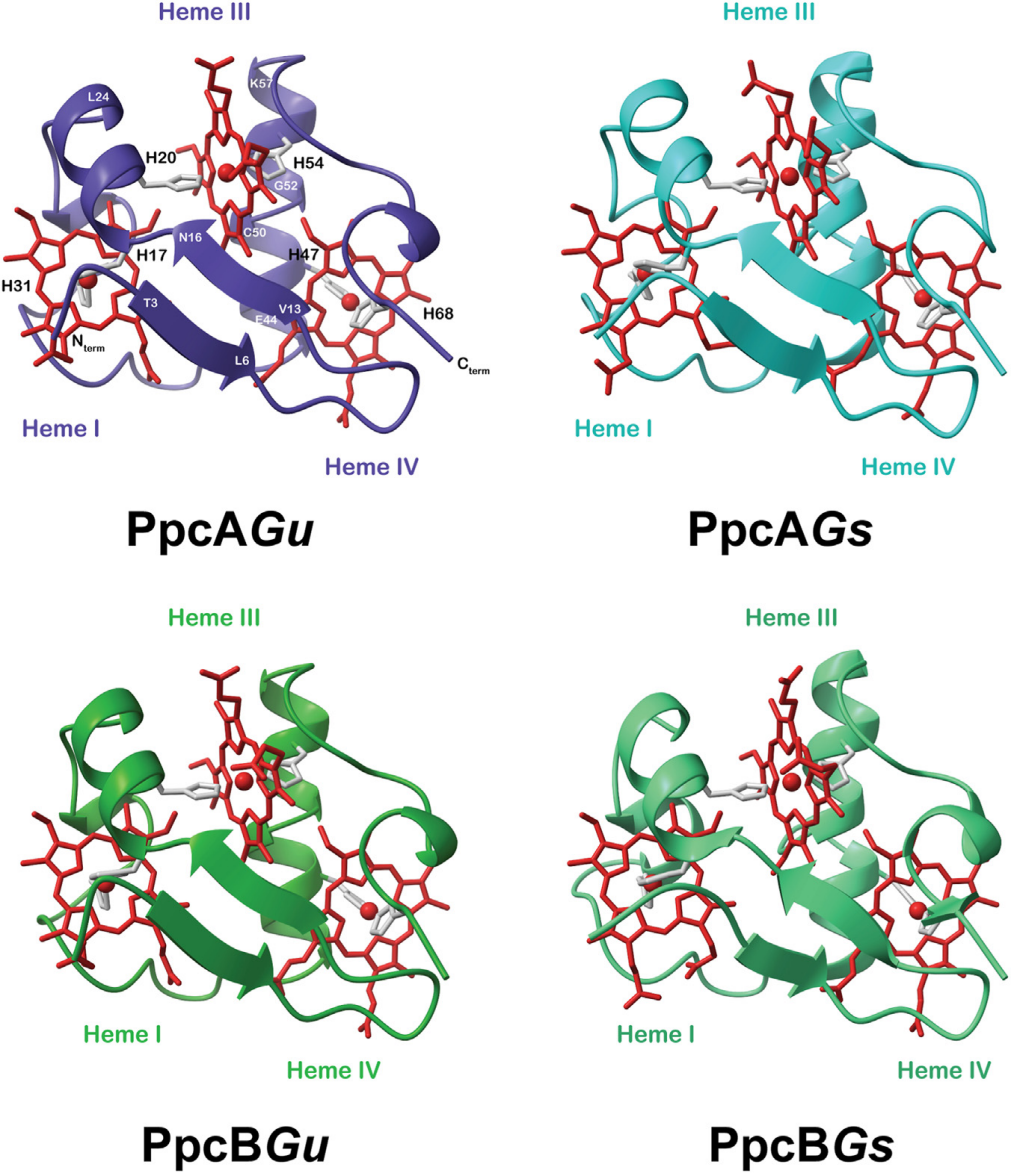
**Three-dimensional structures of PpcA and PpcB from *Geotalea uraniireducens* and *Geobacter sulfurreducens*.** The AlphaFold3 models for PpcA*Gu* and PpcB*Gu* structures are shown as *dark blue* and *dark green* ribbons. The PpcA*Gs* (PDB ID: 2LDO (39)) and PpcB*Gs* (PDB ID: 3BXU (42)) structures are shown as *light blue* and *light green* ribbons. In each model, the heme axial ligands are represented as *gray sticks* and the heme groups are colored in *red*. As a guide, the amino acid numbering is indicated in the PpcA*Gu* model. The pLDDT coloring of the AlphaFold models can be found in Fig. S3. The structures were rendered with ChimeraX (71).

The entire PpcA-family of triheme cytochromes from *G. sulfurreducens* has been characterized in detail by X-ray crystallography and NMR spectroscopy (25, 39, 40, 41, 42). These proteins exhibit a remarkably low amino acid-to-heme ratio (approximately 20 amino acids per heme). This, along with the conservation of the heme-binding motifs in their sequences, contributes to the global maintenance of the heme core and overall fold, which is mainly composed of random coil regions, and a two-strand β-sheet in the N-terminus, followed by several α-helical segments (Fig. 3, right panels). A very similar arrangement is predicted by the AlphaFold3 algorithm (43) for PpcA*Gu* and PpcB*Gu* (Fig. 3, left panels). Overall, the secondary structural motifs and the global arrangement of these cytochromes are predicted with high confidence (pLDDT > 90), except for the N- and C-termini, which are more flexible (Fig. S3).

**Figure 4:**
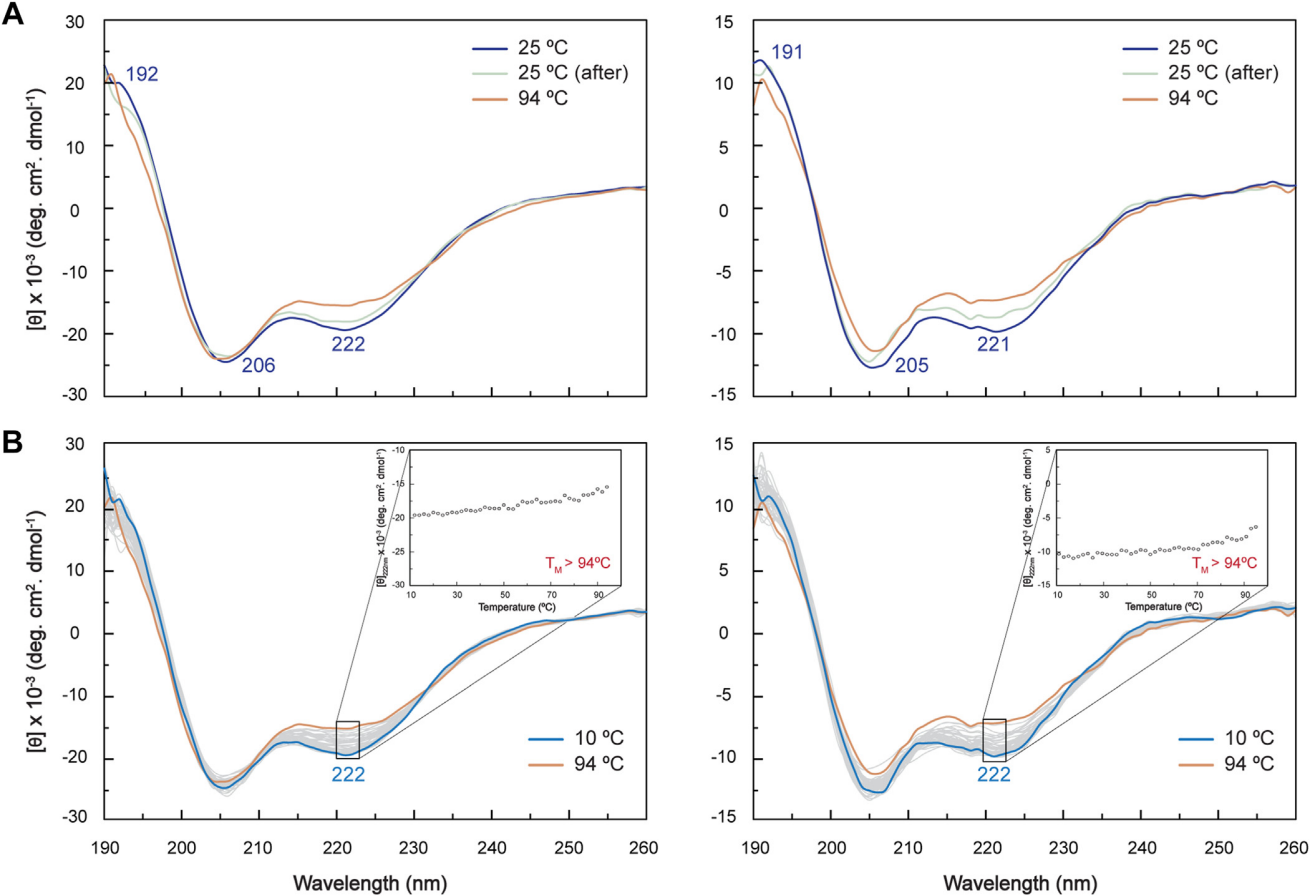
**Far-UV CD spectra of PpcA*Gu* (*left panels*) and PpcB*Gu* (*right panels*) in the oxidized state.***A*, Spectra of PpcA*Gu* (10 μM) and PpcB*Gu* (5 μM) in 10 mM sodium phosphate, pH 8 at 25 °C before (*dark blue*) and after (*gray*) the temperature ramp and at 94 °C (*orange*). In each spectrum, the local maxima and minima are labeled. *B*, thermal stability monitoring of both cytochromes acquired with a temperature range between 10 °C (*blue*) and 94 °C (*orange*). The remaining spectra are represented in *gray*. The thermal unfolding profile of each protein at 222 nm is also represented in the inset panels. “TM” stands for melting temperature.

To validate the secondary structure predictions by AlphaFold3, the CD spectroscopic features of both cytochromes were analyzed in the oxidized state. The CD spectra acquired in the far-UV region (190–260 nm) are shown in Figure 4*A*. Both spectra show a maximum at ∼190 nm (192 and 191 nm for PpcA*Gu* and PpcB*Gu*, respectively) and two minima at ∼208 and ∼222 nm (206 and 222 nm for PpcA*Gu* and 205 and 221 nm for PpcB*Gu*). These features are typical of proteins with a high α-helical content, as further confirmed by the θ_208nm_/θ_222nm_ ratio value (∼1.2) and are in line with the structural models (Fig. 3). The conformational stability of both proteins was investigated through temperature-induced denaturation from 10 to 94 °C. The results demonstrate that both proteins are extremely stable as their secondary structure is essentially unaffected after heating up to 94 °C (see orange spectrum and inset in Fig. 4*B*). After cooling down to 25 °C the original spectral features are recovered (see light gray line in Fig. 4*A*). The high thermostability of the two cytochromes is comparable to that of their homologs in *G. sulfurreducens* (41) and is explained by the spatial arrangement of the three covalently linked heme groups, which are shielded by relatively short polypeptide segments, increasing their local structural stability and integrity (41).

**Figure 5:**
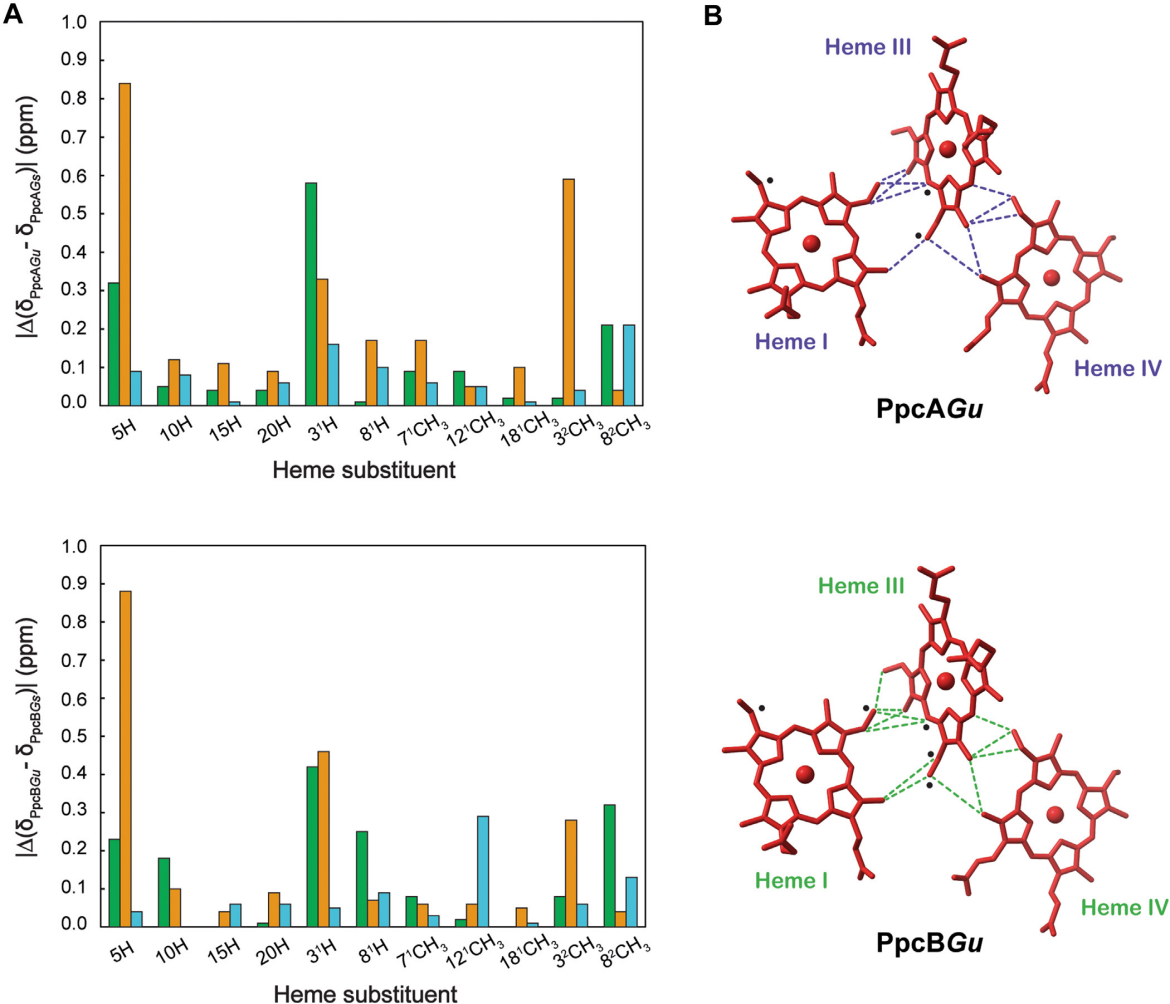
**Heme core architecture of PpcA*Gu* and PpcB*Gu* and respective homologs from *Geobacter sulfurreducens*.***A*, Differences between the ^1^H heme substituents' chemical shifts of PpcA*Gu* and PpcB*Gu* (this work) and those from *G. sulfurreducens* (36, 74) at pH 8 and 15 °C. *Green*, *orange*, and *blue* bars correspond to hemes I, III, and IV, respectively. The RMSDs between the heme proton chemical shifts measured for hemes I, III, and IV are 0.21, 0.33, and 0.09 ppm, respectively, for PpcA and 0.19, 0.31, and 0.10 ppm for PpcB. *B*, Interheme NOE connectivities observed in the 2D ^1^H-NOESY spectra of PpcA*Gu* and PpcB*Gu* (*dashed lines*). The most affected protons are highlighted with *black circles*.

Furthermore, to experimentally confirm that the global fold and the heme core architecture of PpcA*Gu* and PpcB*Gu* are conserved, the ^1^H-NMR signals of the heme groups in the reduced state were specifically assigned (Table S2), as previously described for the *G. sulfurreducens* homologs (42). The small size of the proteins, combined with their low amino acid residues per heme group ratio, make their heme proton signals excellent probes for monitoring structural variations in the heme core architecture. Therefore, the heme ^1^H chemical shifts of cytochromes PpcA*Gu* and PpcB*Gu* were compared to those previously determined for the *G. sulfurreducens* homologs (Fig. 5*A*). Overall, the differences are very small (less than 0.3 ppm), except for one of the edges of heme I and III (see black circles in Fig. 5*B*). These variations correlate with the low global RMSD values (0.23 and 0.22 ppm for PpcA and PpcB, respectively) indicating that the heme cores are conserved in the *G. uraniireducens* cytochromes. This was further confirmed by the analysis of the interheme NOE connectivities observed in the 2D ^1^H-NOESY spectra (see dashed lines in Fig. 5*B*). The analysis of the RMSD values for the individual hemes showed that hemes I and III had slightly higher values than those of heme IV, correlating with the high number of replacements in their vicinity (*cf.* Figs. 1 and 3).

### Screening the order of oxidation of the hemes in *PpcA*Gu and *PpcB*Gu at pH 7

Given the conservation of the heme core and the homology among the cytochromes, a preliminary screening of the individual heme oxidation profiles at pH 7 was conducted before an in-depth thermodynamic characterization of both PpcA*Gu* and PpcB*Gu*. As previously described for the *G. sulfurreducens* homologs (hereafter designated PpcA*Gs* and PpcB*Gs*), triheme cytochromes exhibit three consecutive and reversible steps of one-electron transfer, converting the fully reduced state into the fully oxidized state. This establishes four different redox stages, numbered from *0* (fully reduced) to *3* (fully oxidized), each containing the microstates with the same number of oxidized hemes (Fig. S1). Since electron exchange between hemes within a molecule is fast on the NMR time scale but intermolecular exchange is slow, each stage yields a single set of signals. The chemical shifts of the heme substituents, particularly those of the heme methyl groups, can be traced in each redox stage with 2D ^1^H-EXSY spectra and hence used to probe the oxidation pattern of each heme. Therefore, the assignment of the heme methyl signals in both fully reduced proteins (Table S2) was used to monitor their chemical shift variation up to their final positions in the fully oxidized state as observed in the 2D ^1^H-EXSY spectra (Fig. S4). The assignment of the heme methyl signals in the fully oxidized state (Table S3) was further and independently confirmed through complementary analysis of 2D ^1^H-TOCSY, and 2D ^1^H,^13^C-HMQC spectra (Fig. S5), following a strategy previously described for the *G. sulfurreducens* homologs (44).

**Figure 6:**
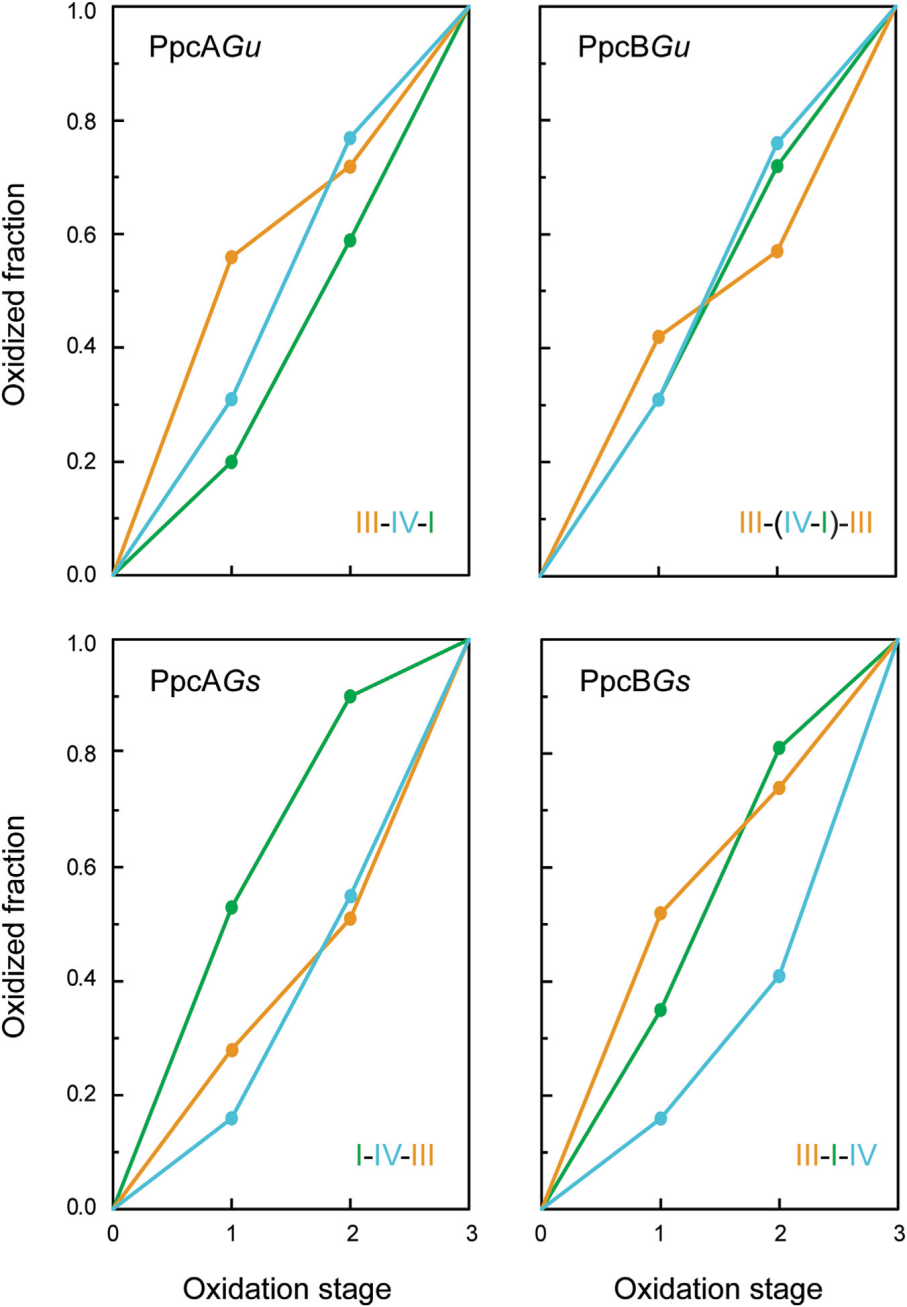
**Heme oxidation fraction values of cytochromes PpcA*Gu* and PpcB*Gu* and their homologs from *Geobacter sulfurreducens* at pH 7.***Green*, *orange*, and *blue colors* represent hemes I, III, and IV, respectively. The heme oxidation fractions for the *Geotalea uraniireducens* cytochromes are indicated in Table S4.

The chemical shifts of the heme methyl signals 2^1^CH_3_^I^, 12^1^CH_3_^III^, and 18^1^CH_3_^IV^ were used to determine the oxidation profiles of the hemes in PpcA*Gu* and PpcB*Gu* (Table S4). These specific signals were selected because they point outward from the heme core, making them less affected by the oxidation of the neighboring hemes. The oxidation profile of each heme is depicted in Figure 6 and compared to those of their homologs from *G. sulfurreducens*. In the case of PpcA*Gu*, the first oxidation step is dominated by the oxidation of heme III (56%), followed by heme IV in the second step (46%) and heme I in the third step (41%). This contrasts with the observations for PpcA*Gs*, where heme I dominates the first oxidation step and the other two hemes exhibit similar oxidation profiles. Thus, the order of the heme oxidation differs between the two cytochromes, along with variations in their individual heme oxidation profiles (*cf.* left panels in Fig. 6). In the case of PpcB cytochromes, the first oxidation step is also dominated by the oxidation of heme III, though to a slightly different extent (42% for PpcB*Gu* and 52% for PpcB*Gs*). Beyond this point, the oxidation profiles are considerably different, particularly in the case of heme IV. Indeed, while in PpcB*Gs*, the second and third oxidation steps are dominated by hemes I and IV, respectively, in PpcB*Gu*, these two hemes show nearly identical oxidation profiles (*cf.* right panels in Fig. 6). Even more striking, in PpcB*Gu*, both hemes I and IV play a crucial role in the second oxidation step, resulting in a pattern where heme III, in addition to the first oxidation step, also dominates the last one.

Since both cytochromes from *G. uraniireducens* exhibit significantly distinct oxidation profiles compared to their *G. sulfurreducens* homologs, their redox properties and functional mechanisms were subsequently studied in detail.

### Reduction potential values and redox interaction networks of *PpcA*Gu and *PpcB*Gu

**Figure 7:**
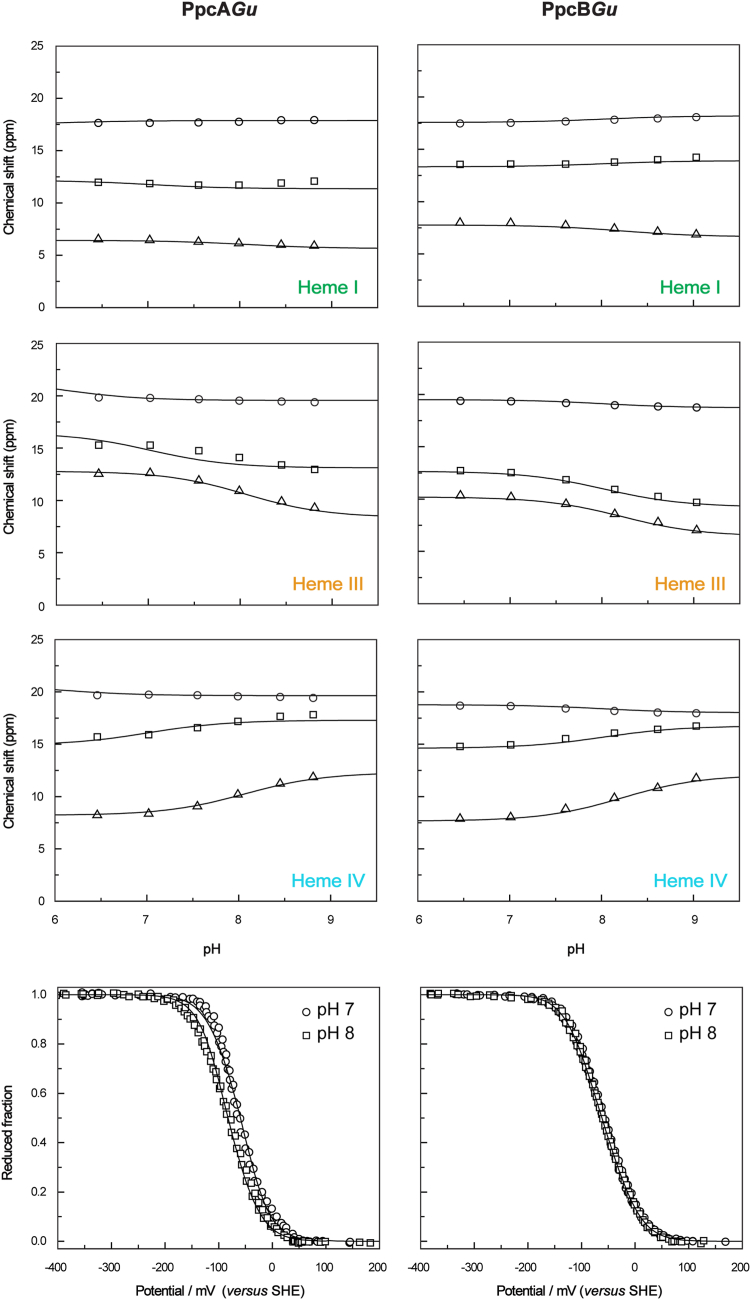
**Fitting of the thermodynamic model to the experimental data of PpcA*Gu* and PpcB*Gu*.** The *black solid lines* indicate the simultaneous fitting of the NMR and UV–visible data. The *upper* panels depict the variation of the heme methyl chemical shifts with the pH at oxidation stages *1* (*triangles*), *2* (*squares*), and *3* (*circles*). The chemical shifts of heme methyls in the fully reduced state (stage *0*) are not indicated since they are unaffected by the pH. The *bottom* panels represent the data points of the redox titrations followed by UV–visible spectroscopy at pH 7 (*circles*) and pH 8 (*squares*).

To quantify the observations discussed above and to determine the redox range in which they occur, 2D ^1^H-EXSY spectra were acquired at several pH values ranging from 6 to 9 for PpcA*Gu* and PpcB*Gu*. The pH dependence of the heme methyl chemical shifts (2^1^CH_3_^I^, 12^1^CH_3_^III^, and 18^1^CH_3_^IV^) measured in the different redox stages, along with data obtained from visible redox titrations at pH 7 and 8, were fitted to the thermodynamic model (Fig. 7). This model, summarized in the Experimental procedures section, was also previously used to determine the detailed thermodynamic properties of the homologous cytochromes PpcA*Gs* and PpcB*Gs* (36). As with these cytochromes, in the case of PpcA*Gu* and PpcB*Gu*, the quality of the fittings demonstrates that the experimental data are well described by the model (Fig. 7). The redox parameters, including heme redox potential values and their redox and redox–Bohr interactions, as well as the p*K*_*a*_ values of the redox-Bohr center across the four oxidation stages, are indicated in Tables 2 and 3, respectively.

**Table 2:** Thermodynamic parameters for the cytochromes PpcA*Gu* and PpcB*Gu* in the fully reduced and protonated form

PpcA*Gu* (this work)	Energy (meV)			
	Heme I	Heme III	Heme IV	Redox-Bohr center
Heme I	**−59 (7)**	28 (4)	17 (5)	−52 (6)
Heme III		**−82 (7)**	32 (4)	−47 (7)
Heme IV			**−66 (8)**	−77 (7)
Redox-Bohr center				**523 (10)**


PpcB*Gu* (this work)	Heme I	Heme III	Heme IV	Redox-Bohr center
Heme I	**−72 (4)**	25 (2)	3 (2)	−2 (4)
Heme III		**−81 (4)**	20 (2)	9 (4)
Heme IV			**−70 (5)**	−29 (4)
Redox-Bohr center				**482 (9)**


PpcA*Gs* (36)	Heme I	Heme III	Heme IV	Redox-Bohr center
Heme I	**−154 (5)**	27 (2)	16 (3)	−32 (4)
Heme III		**−138 (5)**	41 (3)	−31 (4)
Heme IV			**−125 (5)**	−58 (4)
Redox-Bohr center				**495 (8)**


PpcB*Gs* (36)	Heme I	Heme III	Heme IV	Redox-Bohr center
Heme I	**−150 (3)**	17 (2)	8 (2)	−16 (4)
Heme III		**−166 (3)**	32 (2)	−9 (4)
Heme IV			**−125 (3)**	−38 (4)
Redox-Bohr center				**426 (8)**

The diagonal terms (shown in bold) represent the oxidation energies of the three hemes and the deprotonating energy of the redox-Bohr center. The off-diagonal values indicate the redox (heme-heme) and redox-Bohr (heme-proton) interaction energies. All values are reported in meV, with standard errors provided in parentheses. For comparison, the values obtained for PpcA*Gs* and PpcB*Gs* are also indicated.

**Table 3:** Macroscopic p*K*_*a*_ values of the redox-Bohr center in cytochromes PpcA*Gu* and PpcB*Gu*

	Oxidation stage				
Cytochrome	0	1	2	3	Δp*K*_*a*_
PpcA*Gu* (this work)	9.1	8.1	7.0	6.0	3.1
PpcB*Gu* (this work)	8.4	8.2	8.0	8.0	0.4
PpcA*Gs* (36)	8.6	8.0	7.2	6.5	2.1
PpcB*Gs* (36)	7.4	7.1	6.8	6.3	1.1

The values were calculated with the parameters presented in Table 2. The Δp*K*_*a*_ corresponds to the difference between p*K*_*a*_^*red*^ (oxidation stage 0) and p*K*_*a*_^*ox*^ (oxidation stage 3). For comparison, the macroscopic p*K*_*a*_ values obtained for triheme cytochromes PpcA*Gs* and PpcB*Gs* are also indicated.

### Reduction potential values of the hemes

The analysis of Table 2 shows that the reduction potential values of the hemes in the fully reduced and protonated form of PpcA*Gu* are negative and vary: −59, −82, and −66 mV for hemes I, III, and IV, respectively. Compared to its homolog (PpcA*Gs*), these values are less negative (−154, −138, −125 mV for hemes I, III, and IV, respectively). Similarly, in the case of PpcB*Gu*, the reduction potential values of the hemes are also less negative compared to those of PpcB*Gs* (−72, −81, and −70 mV *versus* −150, −166, and −125 mV for hemes I, III, and IV, respectively). In addition to the different redox potential ranges covered by the two pairs of homologous cytochromes, it is noteworthy that the reduction potential values of the hemes in PpcB*Gu* are even more similar than those of PpcA*Gu* (Table 2). This similarity suggests a distinct functional mechanism for the two cytochromes.

Extensive and detailed studies on mutated forms of PpcA*Gs* have shown that the reduction potential values of the hemes are predominantly influenced by the charge of nearby residues and alterations in the hemes’ solvent exposure (37, 45, 46, 47, 48, 49, 50, 51). The amino acid sequences of the two PpcA cytochromes indicate that they are very similar in the number of negatively and positively charged residues (Fig. 1). Specifically, PpcA*Gu* and PpcA*Gs* differ by 16 non-conserved residues, plus one inserted glycine at position 47 in the latter protein. Overall, PpcA*Gu* has three fewer positive charges and two more negative ones. While this would generally suggest a stabilization of the oxidized form and a decrease in the reduction potential values, this is not observed. However, pinpointing the exact locations of the replacements within the protein structure is much more relevant than this broad analysis. In fact, the two-strand β-sheet at the N-terminus formed by Asp^3^-Lys^9^ and Asn^10^-His^17^ are placed in the vicinity of hemes I and III. In this region, PpcA*Gu* has two fewer negative charges (Thr^3^ and Asn^12^ in PpcA*Gu versus* Asp^3^ and Asp^12^ in PpcA*Gs*), which most likely contribute to the stabilization of the reduced form of the hemes and hence to their higher reduction potential values (Table 2). On the other hand, the deletion of one residue (Gly^47^ - PpcA*Gs* numbering) in the long α-helix placed between hemes III and IV brings Lys^48^ and Lys^51^ closer to heme IV in PpcA*Gu*, which might explain its higher reduction potential value (Table 2). This last feature is also observed in the case of PpcB*Gu*, where the presence of lysine residues close to heme I (Lys^25^ and Lys^28^) and III (Lys^57^) also contribute to the higher reduction potential values of these hemes.

### Modulation of the heme reduction potential values by redox interactions

The redox interactions between the hemes measure the effect of the oxidation state of one heme on the reduction potential, of its neighbors, and their magnitude correlates with the distances between the heme iron atoms (52). Therefore, given the highly conserved heme core within the studied triheme cytochromes (Fig. 5), the heme–heme redox interactions are expected to follow a similar pattern. Indeed, this was observed for PpcA*Gu* and PpcB*Gu*, which exhibit larger redox interactions between the closest pairs of hemes (I-III and III-IV), confirming that the redox properties are modulated during the protein oxidation (Table 2). The positive values indicate that the oxidation of a particular heme stabilizes the reduced form of a neighboring heme by increasing its reduction potential value. This means that the heme–heme redox interactions are critical in establishing a specific order of oxidation of the heme groups. Since the heme–heme redox interactions are comparable for the cytochromes from *G. uraniireducens* and *G. sulfurreducens*, the variations are likely attributable to slight differences in iron-iron distances and the amino acid composition surrounding the heme groups.

For the fully reduced and protonated form of PpcA*Gu*, the first oxidation step is dominated by the oxidation of heme III (Table 2). Since the reduction potential value of heme IV is slightly more negative than that of heme I and the redox interactions between heme III and these two hemes are similar (28 and 32 mV for heme I and IV, respectively), the second oxidation step is marginally dominated by the oxidation of heme IV followed by heme I in the last oxidation step, yielding the following order of oxidation of the heme groups: III-IV-I. On the other hand, in the case of PpcB*Gu*, the similar reduction potential values of hemes I and IV, as well as their redox interactions with heme III, explain why the first step of oxidation is dominated by the oxidation of this heme, while the subsequent steps are dominated by the oxidation of both heme I and IV to a similar extent.

Considering the homologous cytochromes in *G. sulfurreducens*, distinct oxidation patterns are observed. In PpcA*Gs*, the first oxidation step is clearly dominated by the oxidation of heme I, while in PpcB*Gs*, the oxidation of heme IV dominates the last step. This demonstrates that the order of oxidation of the hemes is clearly distinct for each pair of homologous cytochromes.

### Modulation of the heme reduction potential values by the pH (redox–Bohr interactions)

**Figure 8:**
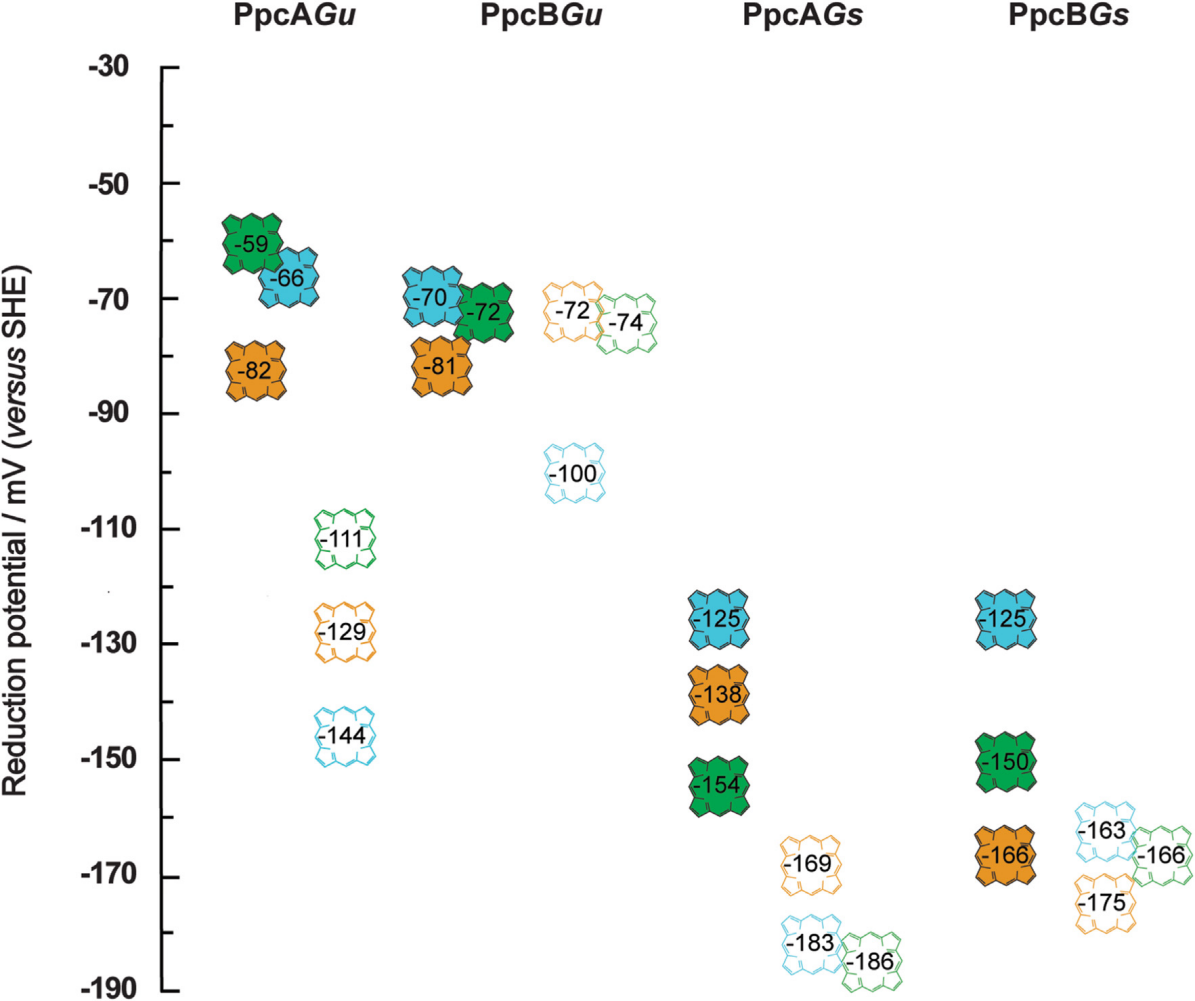
**Heme reduction potential values for PpcA and PpcB cytochromes from *Geotalea uraniireducens* and *Geobacter sulfurreducens* in the reduced protonated (filled hemes) and deprotonated (open hemes) forms.** The reduction potential values for the reduced protonated forms were directly taken from Table 2. The values for the deprotonated forms were obtained by adding the redox–Bohr interactions to the heme reduction potential values of the protonated form (see Table 2).

The thermodynamic parameters listed in Table 2 also highlight the non-negligible influence of the redox–Bohr interactions on the cytochromes from *G. uraniireducens*, indicating that the reduction potential values of the hemes are also modulated by the pH. This effect is illustrated in Figure 8 by comparing the reduction potential values for the fully reduced and protonated cytochromes with those of the deprotonated forms, showing how pH also modulates the stepwise oxidation of the hemes.

For both cytochromes(PpcA*Gu* and PpcB*Gu*), the largest redox–Bohr interactions are observed for heme IV, a feature also observed for their homologs from *G. sulfurreducens* (Table 2). In these cytochromes, the redox-Bohr center was previously assigned to a propionate of heme IV - P_13_^IV^ (39). From a simple electrostatic view, the negative values of the redox–Bohr interactions indicate that deprotonation of this center stabilizes the oxidized form of the hemes by removing of a positive charge in their vicinity. As for the *G. sulfurreducens* homologs, the redox-Bohr effect is considerably higher for PpcA*Gu* than for PpcB*Gu*. This observation is also confirmed by the analysis of the visible redox titration curves obtained at pH 7 and 8 (Fig. 7) and from the total change of the p*K*_*a*_ values (Δp*K*_*a*_ - see Table 3), where the largest variation is observed for PpcA*Gu*.

A larger redox-Bohr effect, as seen in PpcA*Gu*, indicates that the cytochrome has the necessary properties to couple electron/proton transfer, a feature that is functionally relevant if observed within a physiological pH range (see below).

### Heme oxidation profiles of *PpcA*Gu and *PpcB*Gu in the physiological pH range

**Figure 9:**
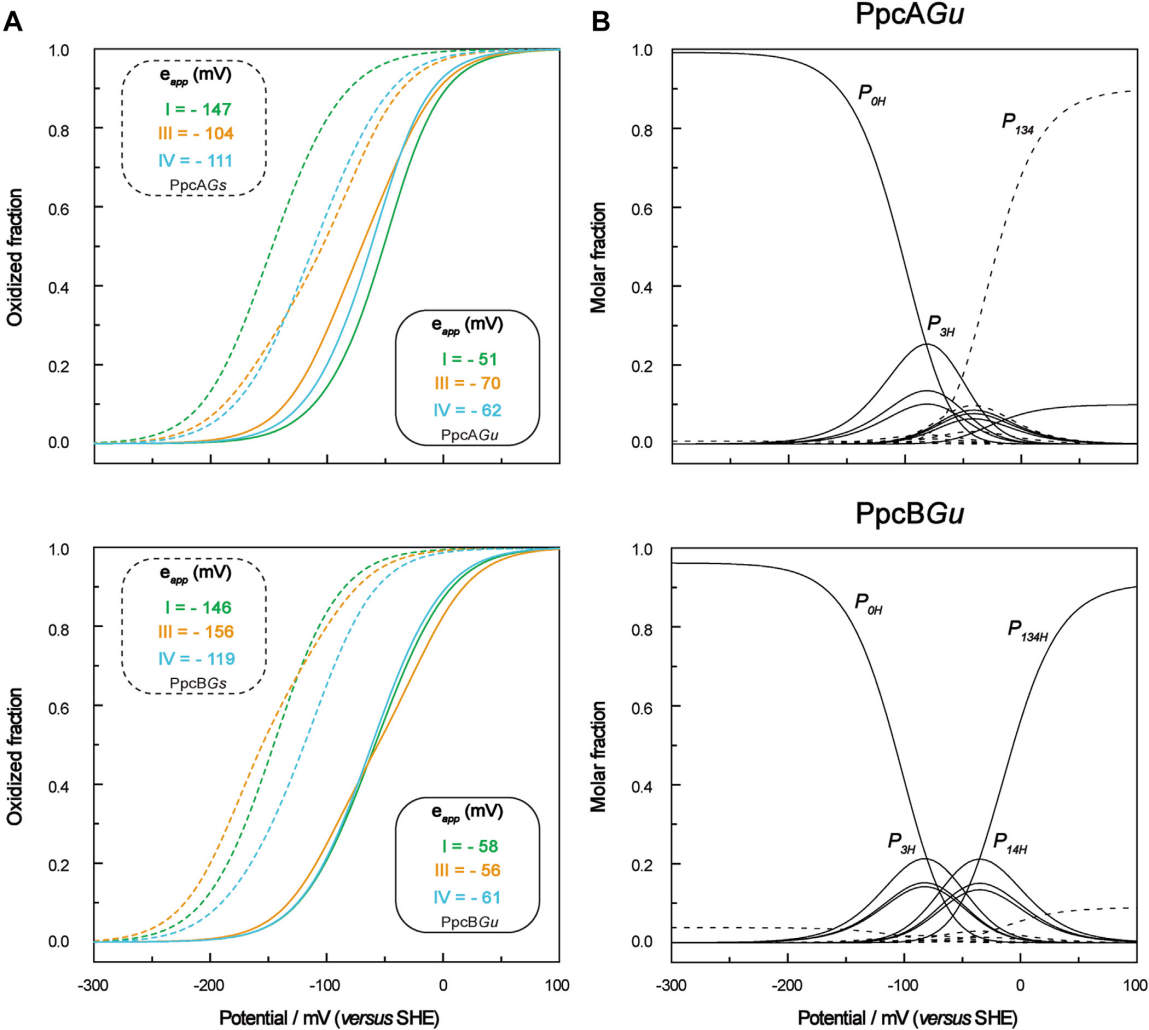
**Comparison of the redox properties of PpcA*Gu* and PpcB*Gu* and respective homologs proteins in *Geobacter sulfurreducens* at pH 7.***A*, Individual heme redox profiles of the *Geotalea uraniireducens* (*solid lines*) and *Geobacter sulfurreducens* (*dashed lines*) cytochromes. The *green*, *orange*, and *blue curves* correspond to hemes I, III, and IV, respectively. The curves were calculated as a function of the solution potential (relative to SHE) using the thermodynamic parameters indicated in Table 2. The microscopic reduction potential values (*e*_*app*_) of each heme in the different cytochromes are indicated in the *solid* and *dashed* insets, respectively. For comparison, the values obtained for PpcA*Gs* and PpcB*Gs* are also indicated (75). *B*, Molar fractions of each microstate of PpcA*Gu* and PpcB*Gu* at pH 7. The molar fractions were determined using the thermodynamic parameters indicated in Table 2. The *solid* and *dashed* lines indicate, respectively, the protonated and deprotonated microstates (see Fig. S1). For clarity, only the curves of the most relevant microstates are labeled.

As mentioned above, the reduction potential value of each heme group is modulated by a precise network of redox and redox–Bohr interactions. Therefore, the thermodynamic parameters (Table 2) can be used to describe the redox behavior of each heme at physiological pH (Fig. 9*A*). The redox behavior of the *G. sulfurreducens* cytochromes has been previously described and is included in this figure for comparison (see dashed lines in Fig. 9*A*). For each pair of homologs, the functional range of the *G. uraniireducens* cytochromes is shifted to less negative values and the separation of the redox potential values is smaller: 19 *versus* 43 mV for PpcA and 5 *versus* 37 mV for PpcB. In addition, as noted for the fully reduced and protonated proteins, the order of oxidation of the hemes is also distinct. Due to the redox–Bohr interactions, these differences are narrowed at physiological pH, particularly in the case of PpcB*Gu* (*cf.* Table 2 and Fig. 9*A*). As a result, the oxidation profiles of the hemes in *G. uraniireducens* cytochromes exhibit unprecedented features compared to any of the periplasmic triheme cytochromes from *Geobacter* bacteria characterized so far (33, 34, 36). In fact, due to the small separation of the reduction potential values, the effect of the redox interactions is more notorious in the oxidation curves of the hemes, explaining the several crossovers observed. In the case of PpcA*Gu*, at low redox potential, heme III dominates the first step of oxidation (Figs. 6 and 9*A*). While heme III oxidizes, it progressively delays the oxidation of the two neighboring hemes due to the positive redox interactions (28 and 32 mV with heme I and IV, respectively). Consequently, the reciprocal effect of these redox interactions on the oxidation profile of heme III is only observed at higher reduction potential values when hemes I and IV are substantially oxidized (see crossover between the orange and blue lines in Fig. 9*A*). An even more remarkable feature is observed in the heme oxidation profiles of PpcB*Gu*. In this case, in the first step of oxidation (low redox potential region), the oxidation curves of all hemes are even more similar (Fig. 6). Since the total value for the redox interactions with heme III is approximately twice as high as for the other two hemes (45 compared to 28 and 23 mV for hemes I and IV, respectively - see Table 2), the reduction potential of heme III is progressively shifted to higher values, such that it also dominates the last step of oxidation, *i.e.*, at high redox potential. This effect explains the crossover observed between the curve of heme III and those of the other hemes (*cf.* Figs. 6 and 9*A*). Overall, the described oxidation profiles for PpcA*Gu* and PpcB*Gu* differ from those of the *G. sulfurreducens* homologs, suggesting that the proteins have distinct functional mechanisms.

### Functional mechanism of *PpcA*Gu and *PpcB*Gu at physiological pH

To elucidate the effect of the redox interaction networks on the functional mechanism of PpcA*Gu* and PpcB*Gu*, the fractional contributions of the 16 microstates (Fig. S1) were determined (Fig. 9*B*). The figure shows significant differences between the relevant microstates for the two cytochromes. In the case of PpcA*Gu*, the oxidation stages *0* and *1* are dominated by the protonated microstates *P*_*0H*_ and *P*_*3H*_, respectively, while the redox-Bohr center is protonated. Interestingly, the stage *2* microstates are surpassed by the *P*_*134*_ curve, which intersects the *P*_*3H*_ curve earlier. Therefore, stage *2* is dominated by the oxidation of hemes I and IV with the concomitant deprotonation of the redox-Bohr center, suggesting a two electron step coupled to proton transfer. Thus, at pH 7, the following route is defined: *P*_*0H*_ → *P*_*3H*_ → *P*_*134*_. The p*K*_*a*_^*red*^ (8.1) and p*K*_*a*_^*ox*^ (6.0) values of the redox-Bohr center encompass the physiological pH range for cellular growth, making the redox-Bohr effect functionally relevant. Compared to PpcA*Gu*, PpcA*Gs* showed a different functional mechanism: *P*_*0H*_ → *P*_*1H*_ → *P*_*14*_ → *P*_*134*_ (36). Although PpcA*Gs* also have the necessary properties to perform a concerted electron/proton transfer, it involves a single electron transfer between oxidation stages *1* and *2* (36).

On the other hand, PpcB*Gu* is unable to perform a concerted electron/proton transfer mechanism at pH 7. In fact, due to the small redox-Bohr effect (Δp*K*_*a*_ = 0.4 - see Table 3), the protonation/deprotonation of the redox-Bohr center coupled to electron transfer will be observable only in a narrow pH range within the *pK*_*a*_^*red*^ (8.2) and *pK*_*a*_^*ox*^ (8.0) values. Nevertheless, a well-defined preferential electron transfer pathway is established: *P0H → P3H → P14H → P134H*. In this case, all the oxidation stages are dominated by a single protonated microstate. Regarding PpcB*Gs*, no preferential electron transfer pathway was defined (36).

## Conclusions

The bacteria *G. uraniireducens* are typically present in subsurface environments, including uranium-contaminated sites, where they play a role in reducing the uranyl cation (UO^2+^), a stable and soluble form of hexavalent uranium (U(VI)), to less soluble tetravalent species (U(IV)), thereby preventing its spread in groundwater (53). This reduction typically occurs at the outer surface of the cell, but uranium reduction has also been detected in the periplasmic space. In this case, periplasmic cytochromes appear to be crucial, as recently shown in the bacterium *G. sulfurreducens* (54). If uranium traverses the outer membrane, its rapid mineralization becomes necessary since the increased level of periplasmic ions seriously compromises the cellular respiratory activities and cell viability (55). In response to periplasmic mineralization, cells have developed detoxification mechanisms, such as the release of outer membrane vesicles containing the periplasmic minerals (55). Consequently, an optimal synergy between periplasmic reduction and detoxification systems might prevent a significant diversion of metabolic energy for this purpose.

In this work, NMR and visible spectroscopy were used to probe the functional properties of PpcA and PpcB periplasmic triheme cytochromes from *G. uraniireducens* (PpcA*Gu* and PpcB*Gu*). The cytochromes share a conserved heme core and a global fold with their homologs from *G. sulfurreducens*. The NMR signal assignment of the heme methyls for both the fully reduced and oxidized states served as the starting points for thermodynamic studies, which involved monitoring the heme methyl chemical shift variations during protein oxidation at different pH values. These data, along with information from visible redox titrations, allowed the determination of heme reduction potential values, heme interactions, and the properties of the redox-Bohr center located near heme IV. The results obtained showed that the heme reduction potential values of both cytochromes are strongly modulated by redox and redox–Bohr interactions. Compared to their homologs from *G. sulfurrreducens*, PpcA*Gu* and PpcB*Gu* cover a different redox functional range shifted to less negative values. The comparative structural analysis between the cytochromes from *G. uraniireducens* and *G. sulfurreducens* provides further evidence of how *G. uraniireducens* cytochromes might have been naturally shaped to contribute to the adaptation of the microorganism to a particular environment. Indeed, the reduction of uranyl cations by uranyl-binding proteins involves their specific interaction with the negatively charged carboxylate groups from aspartate and/or glutamate residues (56). Previous computational studies reveal that the mechanism of uranium reduction in PpcA*Gs* involves the bidentate U(VI) coordination with Glu^32^, which is close to heme I (57). Recently, it was proposed that two other glutamic acid residues in the vicinity of heme IV (Glu^44^ and Glu^67^) could provide a carboxylate group to bind the metal cation (54). Interestingly, the structural models of PpcA*Gu* and PpcB*Gu* not only showed that such characteristics are maintained in *G. uraniireducens*, but they also contain an additional binding site near heme III (Asp^22^ and Glu^22^ in PpcA*Gu* and *PpcBGu*, respectively). Although the negatively charged residues responsible for binding uranyl cations are expected to lower the reduction potential values of the hemes compared to their homologs in *G. sulfurreducens* this was not observed. Instead, the removal of other negatively charged residues near hemes I and III (in the case of PpcA*Gu*), the introduction of positively charged residues (in the case of PpcB*Gu*), and the deletion of a glycine residue, which allows positively charged residues to approach heme IV in both *G. uraniireducens* cytochromes, provide explanations for the increase in the heme reduction potential values. This apparent counterintuitive observation illustrates indeed a very remarkable adaptation strategy to assure an efficient periplasmic reduction of uranium. In fact, the less negative reduction potential values shown by the heme groups of PpcA*Gu* and PpcB*Gu* confer directionality of electron transfer from the inner membrane cytochromes, favoring the reduction of the periplasmic cytochromes. While the reduced form of PpcA*Gu* and PpcB*Gu* is favored, the reduction potential values of their hemes are still sufficiently low to warrant a full reduction of the U(VI)/U(IV) pair (+280 mV (54)).

Additionally, from all the studied periplasmic cytochromes from *Geobacteraceae* family, PpcA*Gu* and PpcB*Gu* revealed unprecedented mechanisms and modulate their redox properties in a way that all the hemes display very similar reduction potential values at physiological pH. Therefore, in these cytochromes, the existence of uranyl cation-binding sites in the proximity of the three hemes and their similar reduction potential values suggest that the diffusion of the electron acceptor to a unique region of the protein is not necessary, and the reduction of uranium can be efficiently carried out by any of the heme groups. This is an excellent example of how highly homologous proteins are specifically adapted in each microorganism. In the case of *G. uraniireducens* cytochromes, the structural environment of the heme framework has been modified to facilitate the simultaneous binding of uranyl cations while maintaining a similar reducing power for all the hemes.

Overall, these functional adjustments reflect a trade-off between benefit and cost, where the cytochromes’ redox properties have evolved to optimize uranium reduction, albeit at the expense of slightly higher reduction potential values. Understanding the specific mechanisms by which these cytochromes fine-tune their redox properties provides valuable insights into how structural features are tailored to enhance electron transfer and reduction processes. Such insights could guide the development of engineered microbes or biomimetic systems for environmental remediation. By elucidating these mechanisms, this study highlights the importance of redox adaptations in cytochromes, broadening our understanding of microbial survival in heavy metal–contaminated environments and paving the way for innovative bioremediation strategies.

## Experimental procedures

### Cloning of cytochromes *PpcA*Gu and *PpcB*Gu

The sequence of the genes encoding the cytochromes PpcA*Gu* (*Gura_4121*) and PpcB*Gu* (*Gura_3843*), with GenBank Accession Number ABQ28264.1 and ABQ27993.1, respectively, was extracted from the genome of *G. uraniireducens* RF4 available on the Kyoto Encyclopedia of Genes and Genomes database (58), with the accession number T00521. Both genomic DNA sequences were amplified to include the region spanning residues 21 to 90, omitting the signal peptide predicted by SignalP 5.0 software (59). Following the restriction-free cloning approach (60), the genes were subsequently incorporated into the pVA203 vector (35) using Phusion High-Fidelity DNA Polymerase (Thermo Fisher Scientific) and the primers listed in Table S1. TaqDNA polymerase (VWR) was used for colony PCR screening, while the template DNA digestion was performed using DpnI (Thermo Fisher Scientific). The intermediate PCR products and the ultimate plasmids were purified using NZYGelpure and NZYMiniprep kits (NZYTech), respectively. Quantification and purity assessment of PCR products and plasmid DNA were conducted with a NanoDrop spectrophotometer ND-100 (Thermo Fisher Scientific). DNA sequencing was carried out by STAB VIDA.

### Protein expression and purification

*E. coli* BL21 (DE3) cells containing the pEC86 plasmid, which encodes the cytochrome *c* maturation gene cluster *ccmABCDEFGH* and a chloramphenicol resistance gene, were transformed with plasmid pVA203 containing the gene *Gura_4121* or *Gura_3843* and an ampicillin resistance gene (61). The cells were cultured on 2xYT medium supplemented with chloramphenicol (34 μg/ml) and ampicillin (100 μg/ml) and grown at 30 °C until they reached an OD_600nm_ of approximately 1.5. At this point, protein expression was induced with 10 μM of IPTG, and the cultures were allowed to grow overnight at the same temperature. After this period, cells were harvested by centrifugation at 6400 *g* for 20 min, and the periplasmic fraction was isolated by incubation with lysis buffer (100 mM Tris–HCl (pH 8), 0.5 mM EDTA, 20% sucrose, and 0.5 mg/ml lysozyme) for 20 min. The periplasmic fraction was recovered by centrifugation at 14,700*g* for 20 min, and the resultant supernatant was further centrifuged at 44000 *g* for 1 h. Finally, the supernatant was dialyzed twice against 2 × 4.5 L of 10 mM Tris–HCl (pH 8) using a membrane with a molecular exclusion limit of 3.5 kDa.

For the protein purification, two distinct chromatographic steps were performed using an ÄKTA Prime Plus Chromatography System (GE Healthcare). The first step involved a cation-exchange chromatography, in which the dialyzed periplasmic fraction of each cytochrome was loaded separately onto a 2 × 5 ml BioScale Mini UNOsphere S cartridges (Bio-Rad) column, equilibrated with 10 mM Tris–HCl (pH 8). The protein elution was conducted using a 150 ml gradient ranging from 0 to 300 mM NaCl at a flow rate of 1 ml/min. The fractions corresponding to the elution peak of the desired protein were selected and concentrated to 1 ml before being injected into a Hiload 16/60 Superdex 75 molecular exclusion column (GE Healthcare) equilibrated with 100 mM sodium phosphate buffer (pH 8). The protein of interest was eluted at a flow rate of 0.5 ml/min. The protein purity was assessed using BlueSafe (NZYTech) stained SDS-PAGE with 12.5% acrylamide/bis-acrylamide.

The concentration of each cytochrome was determined by measuring the absorbance of their α-band in the reduced forms at 552 nm for PpcA*Gu* and 551 nm for PpcB*Gu*. The molar extinction coefficients used were 96 mM^−1^cm^−1^ for PpcA*Gu* and 97 mM^−1^cm^−1^ for PpcB*Gu* (see above). The UV-visible spectra were recorded for both oxidized and reduced samples within 250 to 750 nm range, using an Evolution 201 spectrophotometer (Thermo Fisher Scientific) and quartz cells (Hellma) with 1 cm of path length. The complete reduction of the samples was achieved by the addition of a small excess of sodium dithionite.

### Protein quantification, heme content, and molar extinction coefficient determination

The concentration of the purified cytochromes PpcA*Gu* and PpcB*Gu* was determined using the Modified Lowry Protein Assay Kit (Thermo Fisher Scientific) with horse heart cytochrome *c* as the protein standard. UV-visible spectra were recorded in the range 250 to 750 nm at room temperature for both oxidized and reduced samples and were used to determine the molar extinction coefficient of the cytochromes at 552 nm (PpcA*Gu*) and 551 nm (PpcB*Gu*), corresponding to the α-band of the proteins in the reduced state. The heme content of each protein was determined by the pyridine hemochromogen assay, measuring the absorbance at 550 nm (using ε_550nm_ = 30.27 mM^−1^cm^−1^ for *c*-type hemes (62)) of a 400 μM solution prepared in 75 mM of NaOH/25% pyridine and reduced by the addition of a small excess of sodium dithionite.

### CD spectroscopy

The CD spectra for the PpcA*Gu* and PpcB*Gu* oxidized samples (10 and 5 μM, respectively) prepared in 10 mM sodium phosphate buffer (pH 8) were conducted in the far-UV range (190–260 nm) with an Applied Photophysics Chirascan qCD spectropolarimeter, equipped with a thermostatic cell support, using a 1 mm path length quartz cuvette (Hellma) with a total volume of 350 μl. The spectra recorded at 25 °C are the average of three scans acquired with a bandwidth and a step-size of 1 nm and 3 s per point, respectively. All spectra were adjusted for buffer contribution and the temperature was maintained within ± 1 °C. The mean residue ellipticity ([θ] (deg.cm^2^.dmol^−1^) plotted as a function of wavelength was used to report the CD data (63, 64, 65). [θ] represents the raw data (in degrees) corrected for the protein solution concentration according to Equation 1, where MRW represents the molecular mass of the protein divided by the number of peptide bonds. The concentration of protein is represented by c (g.ml^−1^) and l is the cell path length (cm).

(1) $$\left[\theta \right]=\frac{\theta \times \text{MRW}}{10\times l\times c}$$

To access the conformational stability, temperature-dependent far-UV spectra were recorded in the temperature range of 10 to 94 °C. Measurements were acquired with a stepped ramp mode of 1 s per point, a temperature increment of 2 °C, and a stabilization period of 1 min between each point. After reaching 94 °C, the sample was quickly cooled down to 25 °C and a final spectrum was acquired.

### Potentiometric redox titrations followed by visible spectroscopy

Redox titrations were conducted inside an anaerobic glovebox (LABstar, MBraun) under an argon atmosphere, maintaining oxygen levels below 0.1 ppm. The temperature of the experiments was kept at 15 °C, using an external circulating bath. The visible spectra were recorded using an Evolution 300 spectrophotometer (Thermo Fisher Scientific), connected to the interior of the anaerobic glovebox *via* fiber optics. Protein samples (10 μM) were prepared in 80 mM sodium phosphate buffer with NaCl (250 mM final ionic strength) at pH 7 and 8. The solution redox potential was measured using a combined Pt/Ag/AgCl electrode (Crison), calibrated before and after each titration, with freshly prepared saturated quinhydrone (Merck) solutions at pH 4 and 7. To ensure equilibrium between the redox centers of the proteins and the working electrode, a mixture of redox mediators (covering a potential range from +21 to −440 mV *versus* SHE at pH 7) was used. As previously described (66), each mediator had a final concentration of approximately 2 μM and the mixture included: gallocyanine (*E*^*0'*^ = +21 mV), methylene blue (*E*^*0'*^ = +11 mV), indigo tetrasulfonate (*E*^*0'*^ = −30 mV), indigo trisulfonate (*E*^*0'*^ = −70 mV), indigo disulfonate (*E*^*0'*^ = −110 mV), 2-hydroxy-1,4-napthoquinone (*E*^*0'*^ = −152 mV), anthraquinone-2,6-disulfonate (*E*^*0'*^ = −184 mV), anthraquinone-2-sulfonate (*E*^*0'*^ = −225 mV), safranine O (*E*^*0'*^ = −280 mV), neutral red (*E*^*0'*^ = −325 mV), benzyl viologen (*E*^*0'*^ = −345 mV), diquat (*E*^*0'*^ = −350 mV), and methyl viologen (*E*^*0'*^ = −440 mV). To examine possible hysteresis and to confirm the reversibility of the redox reactions, each redox titration was conducted in both oxidative and reductive directions, by adding successive amounts of sodium dithionite (reducing agent) and potassium ferricyanide (oxidizing agent), respectively. After each addition, sufficient time was provided for a stable measurement of the solution's redox potential. The experiments were performed two times, and the reduction potentials values (relative to SHE) were determined to be consistently reproducible within a range of ± 2 mV. To exclude the optical contribution of redox mediators, the reduced fraction of both cytochromes was obtained by integrating the area of the α-band (552 and 551 nm for PpcA*Gu* and PpcB*Gu*, respectively) above the line connecting the flanking isosbestic points (542 and 561 nm for PpcA*Gu* and 544 and 563 nm for PpcB*Gu*) (36).

### NMR studies

All the NMR experiments were acquired in a Bruker Avance III 600 MHz spectrometer equipped with a triple-resonance cryoprobe at 15 °C. The residual H_2_O signal was used as an internal secondary reference to calibrate the ^1^H chemical shifts in relation to sodium trimethylsilylpropanesulfonate (DDS, Sigma-Aldrich) at 0 ppm, according to previous descriptions (67). All spectra were acquired with pulse sequences from Bruker’s standard pulse sequence library, processed with TopSpin 3.6.2 (Bruker BioSpin) and analyzed with the software Sparky (TD Goddard and DG Kneller, SPARKY 3, University of California).

#### Sample preparation

The protein samples used to assign the heme substituents’ chemical shifts in both the fully reduced and fully oxidized states were prepared at a concentration of approximately 500 μM in 80 mM sodium phosphate with NaCl (final ionic strength of 250 mM) in deuterated water (^2^H_2_O) at pH 8. The pH values of the samples were confirmed using a glass microelectrode. To obtain fully reduced samples, the tubes were first sealed with a gas-tight serum cap, and air was purged from the samples to prevent possible reoxidation. Reduction was then accomplished by introducing gaseous hydrogen in the presence of catalytic amounts of hydrogenase from *Desulfovibrio vulgaris* (Hildenborough) directly in the NMR tube, as previously described (42). Partially oxidized samples (75 μM), used for EXSY experiments, were prepared in the same buffer in the pH range 6 to 9 and obtained by first replacing the hydrogen from the fully reduced sample with argon and then adding controlled amounts of air into the NMR tube using a Hamilton syringe.

#### Assignment of the NMR signals of the heme substituents in the fully reduced state

To assist the assignment of the heme substituents in the reduced state, 2D ^1^H–TOCSY and 2D ^1^H–NOESY experiments were acquired. The 2D ^1^H–TOCSY spectra were recorded using a mixing time of 60 ms and 128 scans per increment, with a total of 2048 (*t*_*2*_) × 512 (*t*_*1*_) data points, covering a sweep width of 8.4 kHz in the ^1^H dimension. Similarly, the 2D ^1^H–NOESY spectra were acquired at a mixing time of 80 ms, collecting 4096 (*t*_*2*_) × 512 (*t*_*1*_) data points, covering a sweep width of 8.4 kHz in the ^1^H dimension, with 128 scans per increment.

#### Assignment of the NMR signals of the heme substituents in the fully oxidized state

For fully oxidized samples, three different 2D NMR experiments were acquired: 2D ^1^H,^13^C –HMQC, 2D ^1^H–TOCSY, and 2D ^1^H–NOESY. The 2D ^1^H,^13^C –HMQC spectra were recorded with a total of 4096 (*t*_*2*_) × 256 (*t*_*1*_) data points, covering a sweep width of 25.3 kHz in the ^1^H dimension and 52.8 kHz in the ^13^C dimension, with 400 scans per increment. 2D ^1^H–TOCSY and 2D ^1^H-NOESY spectra were recorded with a mixing time of 45 and 80 ms, respectively, using the same data points as for the fully reduced samples, covering a sweep width of 25.3 kHz in the ^1^H dimension, with 164 scans per increment.

#### Monitoring the oxidation profile of the heme groups

The oxidation patterns of the hemes were monitored by acquiring a series of 2D ^1^H-EXSY NMR spectra for partially oxidized samples at different pH values. All spectra were recorded with a mixing time of 25 ms, using 2048 (*t*_*2*_) × 256 (*t*_*1*_) data points and 256 scans per increment, covering a sweep width of 43.4 kHz in the ^1^H dimension. Before and after each 2D spectra, 1D ^1^H NMR spectra were acquired to select the desired partial oxidation level of the samples and to confirm that the oxidation level of the samples remained stable during the acquisition, respectively. The 1D ^1^H-NMR spectrum was acquired with water pre-saturation, collecting 32.8 k data points to cover a spectral width of 26.0 kHz and 128 scans per increment.

### Determination of the thermodynamic parameters

In contrast to monoheme cytochromes, where the reduction potential of the heme can be directly obtained from a potentiometric redox titration followed by visible spectroscopy, this is not possible in multiheme cytochromes due to the coexistence of multiple microstates in solution (36). In the case of a triheme cytochrome, three consecutive reversible steps of one-electron transfer can be defined while the fully reduced state is converted into the fully oxidized state. Thus, four distinct redox stages, numbered *S*_*0*_ to *S*_*3*_, can be defined and categorized according to the identical number of oxidized heme groups (Fig. S1) (36). Additionally, the microstates are distinguished according to the protonation or deprotonation of the redox-Bohr center, leading to a total of 16 possible microstates. The protonation/deprotonation of the redox-Bohr center modulates the heme reduction potential values (redox–Bohr interactions), which are also modulated by heme–heme redox interactions due to the spatial proximity of the heme groups (redox-interactions). Therefore, the detailed characterization of a triheme cytochrome encompasses the determination of 10 parameters: three heme reduction potential values, three redox interactions, the p*K*_*a*_ of the redox-Bohr center, and three redox-Bohr interactions. Once determined, these values can be used to calculate the fractional contribution of each microstate across the full range of pH and solution potential (for a review, see (68)).

Experimentally, the determination of the above-mentioned thermodynamic parameters is achieved by combining data obtained from redox titrations followed by UV-visible spectroscopy and 2D ^1^H–EXSY NMR spectra. In fact, when the electron exchange rate between microstates within the same oxidation stage - intramolecular electron transfer - is fast and the electron exchange rate between microstates from different oxidation stages - intermolecular electron transfer - is slow on the NMR timescale, the signals of the heme substituents have distinct chemical shifts in each oxidation state. Since the variation of the chemical shifts is proportional to the oxidation state of a particular heme group, they can be used to monitor the stepwise oxidation of the hemes from the fully reduced state to the fully oxidized state. The relative information obtained from the NMR redox titrations is then combined with the data obtained from potentiometric redox titrations to obtain the absolute values of the thermodynamic parameters (69, 70).

In the present work, the stepwise oxidation of the hemes was measured by 2D ^1^H–EXSY experiments performed in the pH range of 6 to 9. These data were simultaneously fitted with data obtained from visible redox titrations conducted at pH 7 and 8. The visible data points were assessed to have an error of 3% of the optical signal. In contrast, the experimental uncertainty of the NMR data was calculated based on the linewidth of each NMR signal at half height.

## Data availability

All relevant data are available from the corresponding author upon reasonable request.

## Supporting information

This article contains supporting information (71, 72).

## Conflicts of interest

The authors declare that they have no conflicts of interests with the contents of this article.
