# Supplementary material for: New insights in uranium bioremediation by cytochromes of the bacterium Geotalea uraniireducens
Source: J Biol Chem. 2024 Dec 14;301(2):108090. doi: 10.1016/j.jbc.2024.108090 (PMC11787507; doi:10.1016/j.jbc.2024.108090)
Supplement: Supporting information [file mmc1.docx]

**New insights in uranium bioremediation by cytochromes of bacterium *Geotalea uraniireducens***

Alexandre Almeida^1,2^, David L. Turner^3^, Marta A. Silva^1,2*^, Carlos A. Salgueiro^1,2*^

^1^Associate Laboratory i4HB – Institute for Health and Bioeconomy, NOVA School of Science and Technology, Universidade NOVA de Lisboa, 2819-516 Caparica, Portugal.

^2^UCIBIO – Applied Molecular Biosciences Unit, Chemistry Department, NOVA School of Science and Technology, Universidade NOVA de Lisboa, 2829-516 Caparica, Portugal.

^3^Instituto de Tecnologia Química e Biológica António Xavier, Universidade Nova de Lisboa, Oeiras, Portugal.

***Corresponding authors:** mafs@fct.unl.pt; csalgueiro@fct.unl.pt

**Keywords:** Electrogenic bacteria, Electron transfer, Multiheme *c*-type cytochromes, Redox characterization, Nuclear Magnetic Resonance (NMR)

**Supplementary Information**

**Supplementary Tables**

**Table S1 – Sequences of the primers used to produce the vectors containing the genes for PpcA*Gu* and PpcB*Gu*.** The DNA sequences specific to the plasmid pVA203 are highlighted in blue, whereas those corresponding to the amplified regions of the PpcA*Gu* and PpcB*Gu* genes are highlighted in green. The primers were purchased from Invitrogen. The abbreviations fw and rv stand for forward and reverse, respectively.

|  | | DNA sequence (5´- 3´) |
| --- | --- | --- |
| PpcA*Gu_*fw | **GCTACCGTTGCGGCCGCCGCCGACACCATCACCCTG** | |
| PpcA*Gu*_rv | **AGCTTGTCGACGGAGCTCGAATTTACTTCTTGTGGCACTCACC** | |
| PpcB*Gu_*fw | **GCTACCGTTGCGGCCGCCGCTGATACCATTACCCTGCC** | |
| PpcB*Gu*_rv | **AGCTTGTCGACGGAGCTCGAATTTACTTCTTGTGGCACTCTCC** | |

**Table S2 – ^1^H chemical shifts of the heme substituents from PpcAGu and PpcBGu in the reduced state (pH 8 and 15 °C).** The heme substituents are labeled according to the IUPAC-IUB nomenclature [76].

| Heme substituent | Protein | Chemical shifts (ppm) | | |
| --- | --- | --- | --- | --- |
|  |  | **Heme I** | **Heme III** | **Heme IV** |
| 5H | PpcA*Gu* | 9.33 | 9.74 | 8.93 |
|  | PpcB*Gu* | 9.48 | 9.75 | 8.96 |
| 10H | PpcA*Gu* | 9.07 | 9.74 | 9.25 |
|  | PpcB*Gu* | 9.08 | 9.77 | 9.27 |
| 15H | PpcA*Gu* | 9.30 | 9.56 | 9.50 |
|  | PpcB*Gu* | 9.27 | 9.55 | 9.46 |
| 20H | PpcA*Gu* | 9.54 | 10.05 | 9.45 |
|  | PpcB*Gu* | 9.49 | 10.12 | 9.45 |
| 3^1^H | PpcA*Gu* | 5.72 | 6.58 | 5.88 |
|  | PpcB*Gu* | 5.88 | 6.47 | 5.91 |
| 8^1^H | PpcA*Gu* | 6.28 | 6.43 | 6.18 |
|  | PpcB*Gu* | 6.18 | 6.46 | 6.19 |
| 2^1^CH_3_ | PpcA*Gu* | 3.57 | 4.14 | 3.66 |
|  | PpcB*Gu* | 3.57 | 4.23 | 3.65 |
| 7^1^CH_3_ | PpcA*Gu* | 3.49 | 3.97 | 2.96 |
|  | PpcB*Gu* | 3.57 | 4.08 | 2.98 |
| 12^1^CH_3_ | PpcA*Gu* | 2.64 | 3.45 | 3.90 |
|  | PpcB*Gu* | 2.71 | 3.46 | 3.90 |
| 18^1^CH_3_ | PpcA*Gu* | 3.36 | 3.96 | 3.33 |
|  | PpcB*Gu* | 3.36 | 3.97 | 3.33 |
| 3^2^CH_3_ | PpcA*Gu* | 2.16 | 2.32 | 2.10 |
|  | PpcB*Gu* | 2.09 | 2.18 | 2.10 |
| 8^2^CH_3_ | PpcA*Gu* | 2.00 | 3.02 | 1.34 |
|  | PpcB*Gu* | 1.79 | 3.01 | 1.35 |

**Table S3 –^1^H and ^13^C chemical shifts of heme methyls and propionates groups from PpcA*Gu* and PpcB*Gu* in the oxidized state (pH 8 and 15 °C).**

| Heme substituent |  | Chemical Shifts (ppm) | | | | | |
| --- | --- | --- | --- | --- | --- | --- | --- |
|  | **Protein** | **Heme I** | | **Heme III** | | **Heme IV** | |
|  |  | **^13^C** | **^1^H** | **^13^C** | **^1^H** | **^13^C** | **^1^H** |
| 2 ^1^CH_3_ | PpcA | -37.51 | 17.79 | -29.33 | 12.87 | -26.43 | 10.08 |
|  | PpcB | -37.79 | 17.74 | -27.74 | 12.51 | -28.16 | 11.12 |
| 7 ^1^CH_3_ | PpcA | -20.76 | 7.05 | -33.62 | 12.90 | -32.47 | 15.97 |
|  | PpcB | -20.99 | 7.85 | -32.96 | 13.39 | -30.36 | 14.53 |
| 12 ^1^CH_3_ | PpcA | -56.00 | 23.81 | -35.96 | 19.52 | -30.38 | 11.64 |
|  | PpcB | -55.85 | 23.45 | -34.56 | 19.03 | -32.64 | 12.68 |
| 13 ^1^CH_2_ | PpcA | -17.12 | 1.45 | -59.48 | 18.04 | -15.16 | 0.79 |
|  |  |  | 7.80 |  | 19.42 |  | 3.80 |
|  | PpcB | -14.27 | 1.72 | -59.96 | 17.07 | -16.46 | 2.33 |
|  |  |  | 6.50 |  | 20.53 |  | 3.76 |
| 13 ^2^CH_2_ | PpcA | 92.62 | -1.58 | 169.01 | -2.00 | 85.61 | -1.58 |
|  |  |  | -0.53 |  | -1.16 |  | -1.18 |
|  | PpcB | 88.58 | -1.45 | 169.50 | -1.66 | 87.85 | -1.65 |
|  |  |  | -0.59 |  | -0.99 |  | -1.49 |
| 17 ^1^CH_2_ | PpcA | -6.75 | 0.34 | -14.83 | -2.26 | -24.74 | 6.72 |
|  |  |  | 1.70 |  | 7.54 |  | 7.24 |
|  | PpcB | -8.42 | 0.31 | -14.60 | -1.15 | -23.64 | 5.67 |
|  |  |  | 3.58 |  | 6.27 |  | 7.16 |
| 17 ^2^CH_2_ | PpcA | 72.48 | -1.42 | 85.92 | -3.23 | 109.23 | -0.71 |
|  |  |  | -0.95 |  | 0.33 |  | 0.36 |
|  | PpcB | 76.28 | -0.76 | 86.73 | -2.47 | 106.31 | -0.73 |
|  |  |  | -0.36 |  | 0.09 |  | 0.28 |
| 18 ^1^CH_3_ | PpcA | -39.56 | 15.91 | -4.50 | 0.90 | -44.19 | 19.56 |
|  | PpcB | -40.99 | 17.12 | -2.47 | -0.12 | -42.30 | 18.25 |

**Table S4 – Redox-dependence of the ^1^H heme methyl chemical shifts and heme oxidation fractions of PpcA*Gu* and PpcB*Gu* (pH 7 and 15 °C).** The heme methyls 2^1^CH_3_^I^, 12^1^CH_3_^III^ and 18^1^CH_3_^IV^ were chosen to monitor each heme oxidation through the four different oxidation stages (see main text). The oxidation fractions of the hemes in each oxidation stage (*x_i_*,) were calculated according to the equation *x_i_* = (*δ_i_*-*δ_0_*)/(*δ_3_*-*δ_0_*), where *δ_i_*, *δ_0_*, and *δ_3_* are the observed chemical shifts of the heme methyls in stages *i*, *0* (fully reduced), and *3* (fully oxidized), respectively (see also Fig. S1). The Σ *x_i_* indicates the sum of the oxidation fractions, which value represents the number of oxidized hemes in each oxidation stage.

|  | **Oxidation stage** | |  | **Chemical shift (ppm)** | | |  | ***x_i_*** | | |  | **Σ *x_i_*** |
| --- | --- | --- | --- | --- | --- | --- | --- | --- | --- | --- | --- | --- |
|  | |  |  | **I** | **III** | **IV** |  | **I** | **III** | **IV** |  |  |
| **PpcA*Gu*** | | *0* |  | 3.57 | 3.45 | 3.33 |  | 0 | 0 | 0 |  | 0 |
|  |  | *1* |  | 6.45 | 12.61 | 8.35 |  | 0.20 | 0.56 | 0.31 |  | 1.07 |
|  |  | *2* |  | 11.85 | 15.28 | 15.91 |  | 0.59 | 0.72 | 0.77 |  | 2.08 |
|  |  | *3* |  | 17.65 | 19.80 | 19.75 |  | 1 | 1 | 1 |  | 3 |
| **PpcB*Gu*** | | *0* |  | 3.57 | 3.46 | 3.33 |  | 0 | 0 | 0 |  | 0 |
|  |  | *1* |  | 7.95 | 10.19 | 8.01 |  | 0.31 | 0.42 | 0.31 |  | 1.04 |
|  |  | *2* |  | 13.59 | 12.53 | 14.94 |  | 0.72 | 0.57 | 0.76 |  | 2.05 |
|  |  | *3* |  | 17.53 | 19.35 | 18.65 |  | 1 | 1 | 1 |  | 3 |

**Supplementary Figures**


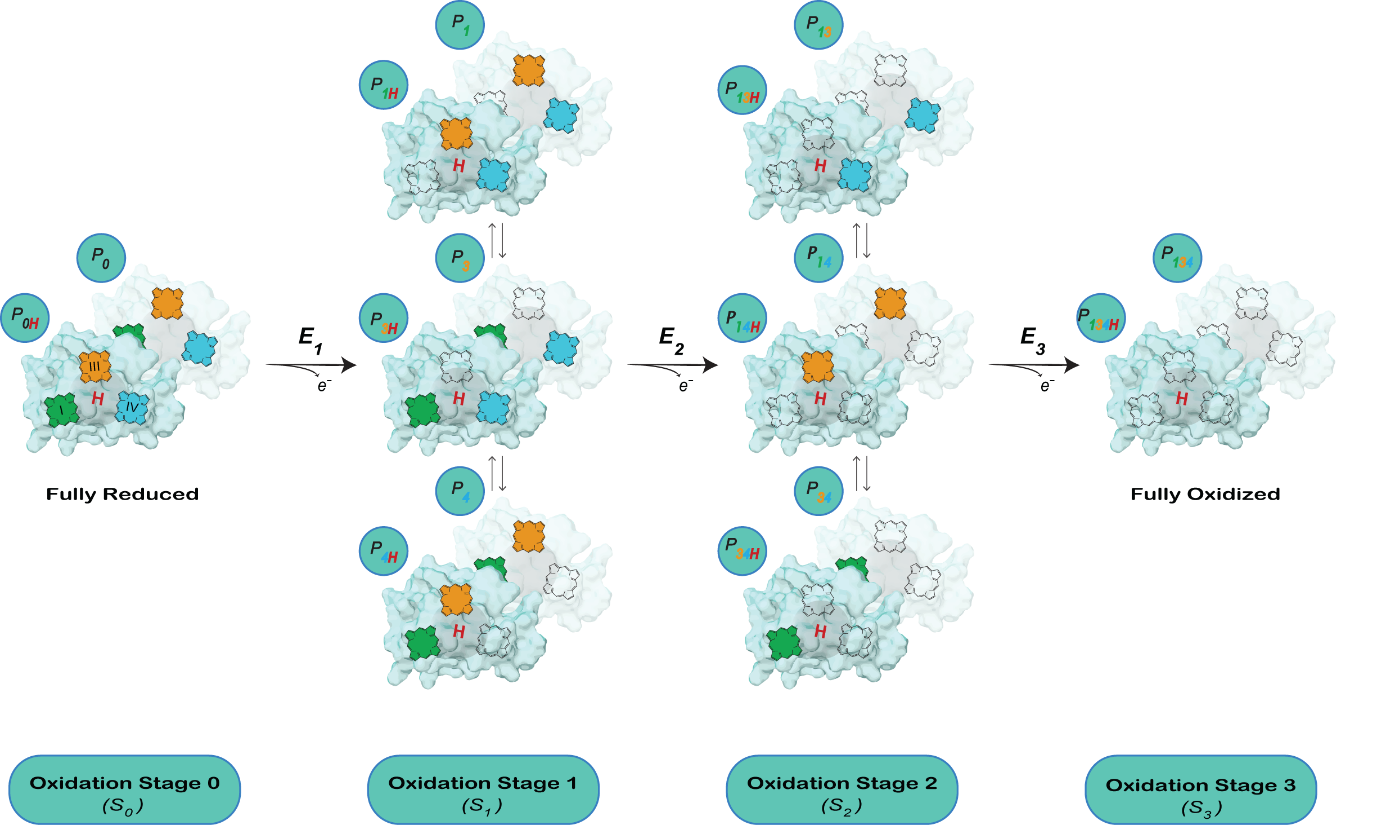


**Figure S1 – Schematic representation of the electronic distribution for a triheme cytochrome featuring one redox-Bohr center and showcasing the 16 possible microstates.** The dark and light protein surfaces represent the protonated and deprotonated microstates, respectively. The protonated microstates are additionally marked with a red acid-base group (H). In each microstate, the heme groups are colored green (heme I), orange (heme III) and blue (heme IV), which can be either in the reduced state (colored) or oxidized state (uncolored). The microstates are arranged into four oxidation stages (S_0_ - S_3_), each containing the same number of oxidized hemes, linked by three one-electron redox steps. E_1_, E_2_ and E_3_ represent the macroscopic reduction potentials values for the first, second and third oxidation steps, respectively. P_0H_ and P_0_ denote the fully reduced protonated and deprotonated microstates, respectively. P_ijkH_ and P_ijk_ indicate the protonated and deprotonated microstates with heme(s) i, j and k oxidized.

**
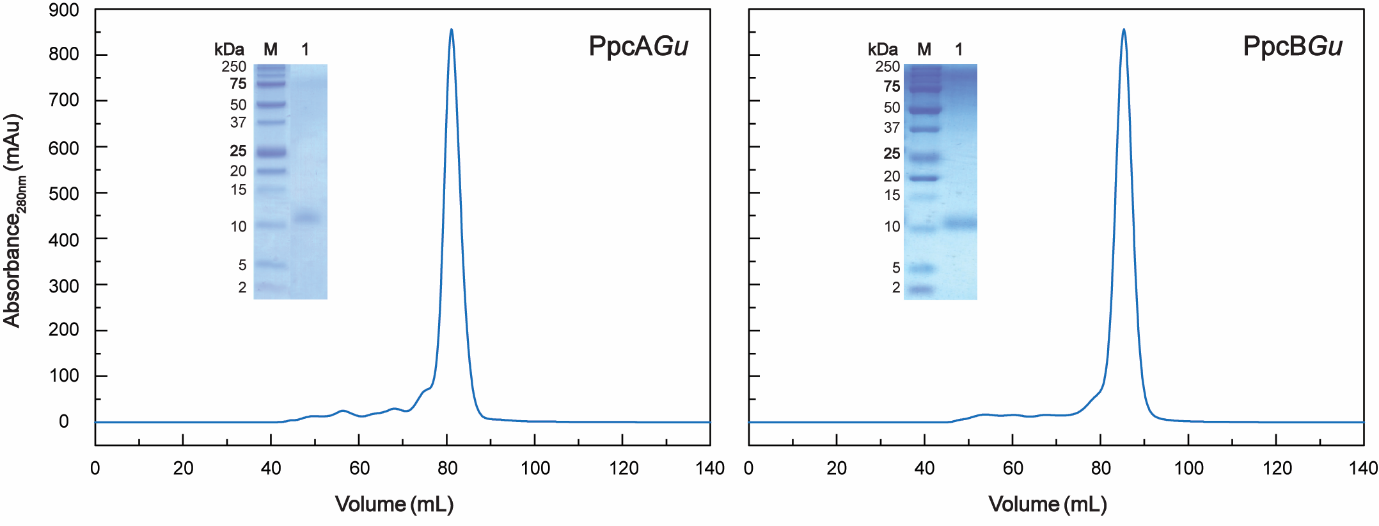
**

**Figure S2 – Size-exclusion chromatography elution profile and SDS-PAGE of purified cytochromes PpcAGu and PpcBGu.** The chromatograms show the elution profiles of the cytochromes in a Hiload 16/60 Superdex 75 molecular exclusion column (GE Healthcare) in 100 mM sodium phosphate buffer, pH 8. The fractions corresponding to pure cytochromes (the most intense peak of the chromatograms) show a single band in the SDS-PAGE gel (lane 1). The molecular weight marker (lane M) used was the Protein Plus Protein^TM^ Dual Xtra Standards (Bio-Rad), and the molecular weights (kDa) are shown on the left.

**
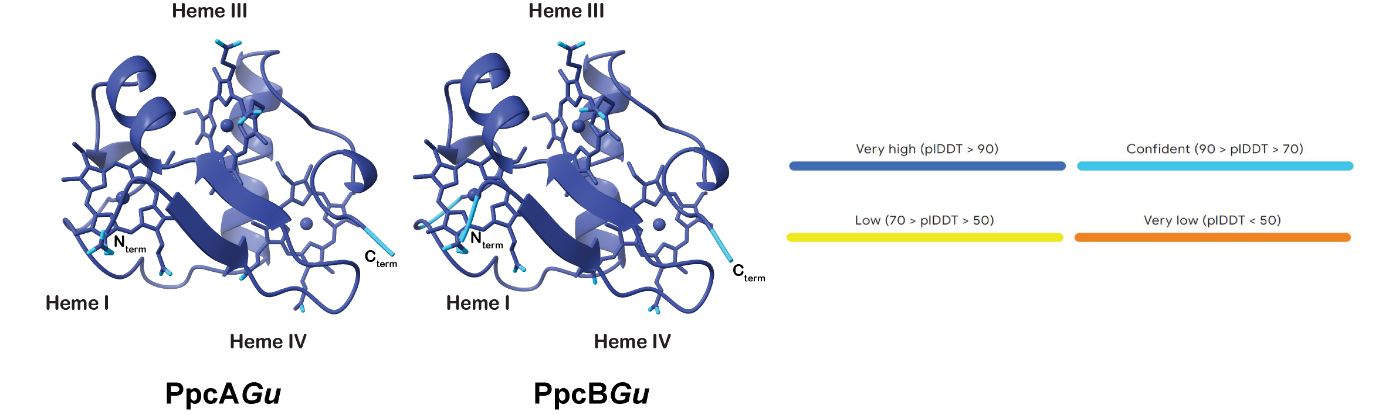
**

**Figure S3 – AlphaFold3 models’ confidence of PpcA*Gu* and PpcB*Gu*.** Both structures are represented as ribbons and colored according to the AlphaFold database predicted local distance difference test (pLDDT) values. The structures were rendered with ChimeraX [72].

**
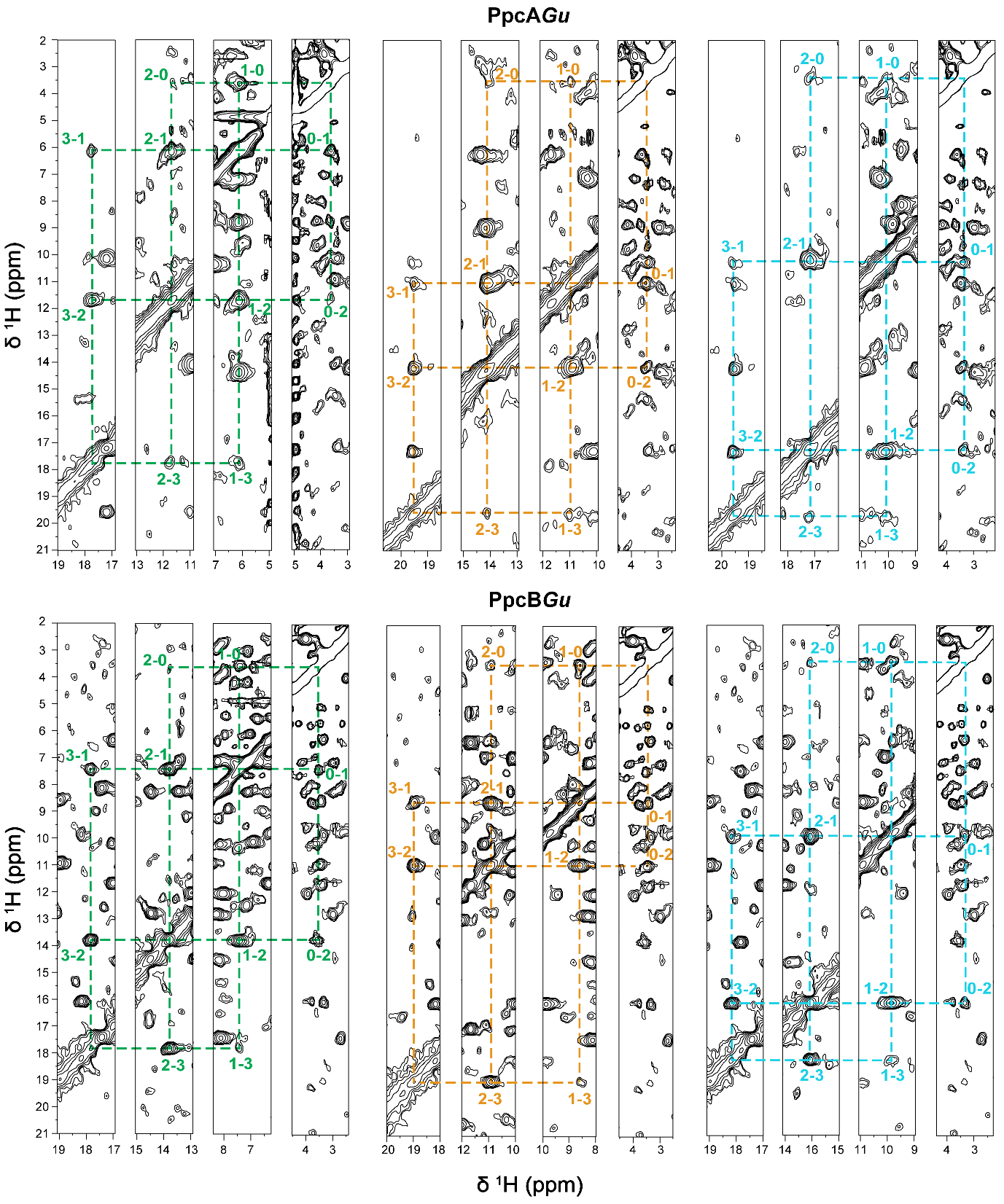
**

**Figure S4 – Heme oxidation profiles of PpcAGu (upper panel) and PpcBGu (bottom panel) at pH 8 and 15 °C.** For each heme, the 4 panels represent expansions of 2D ^1^H-EXSY NMR spectra in the different oxidation stages (see Fig. S1). The cross-peaks resulting from intermolecular electron transfer between the different oxidation stages (0-3) are indicated by dashed lines for heme methyls 2^1^CH_3_^I^ (green), 12^1^CH_3_^III^ (orange), and 18^1^CH_3_^IV^ (blue).


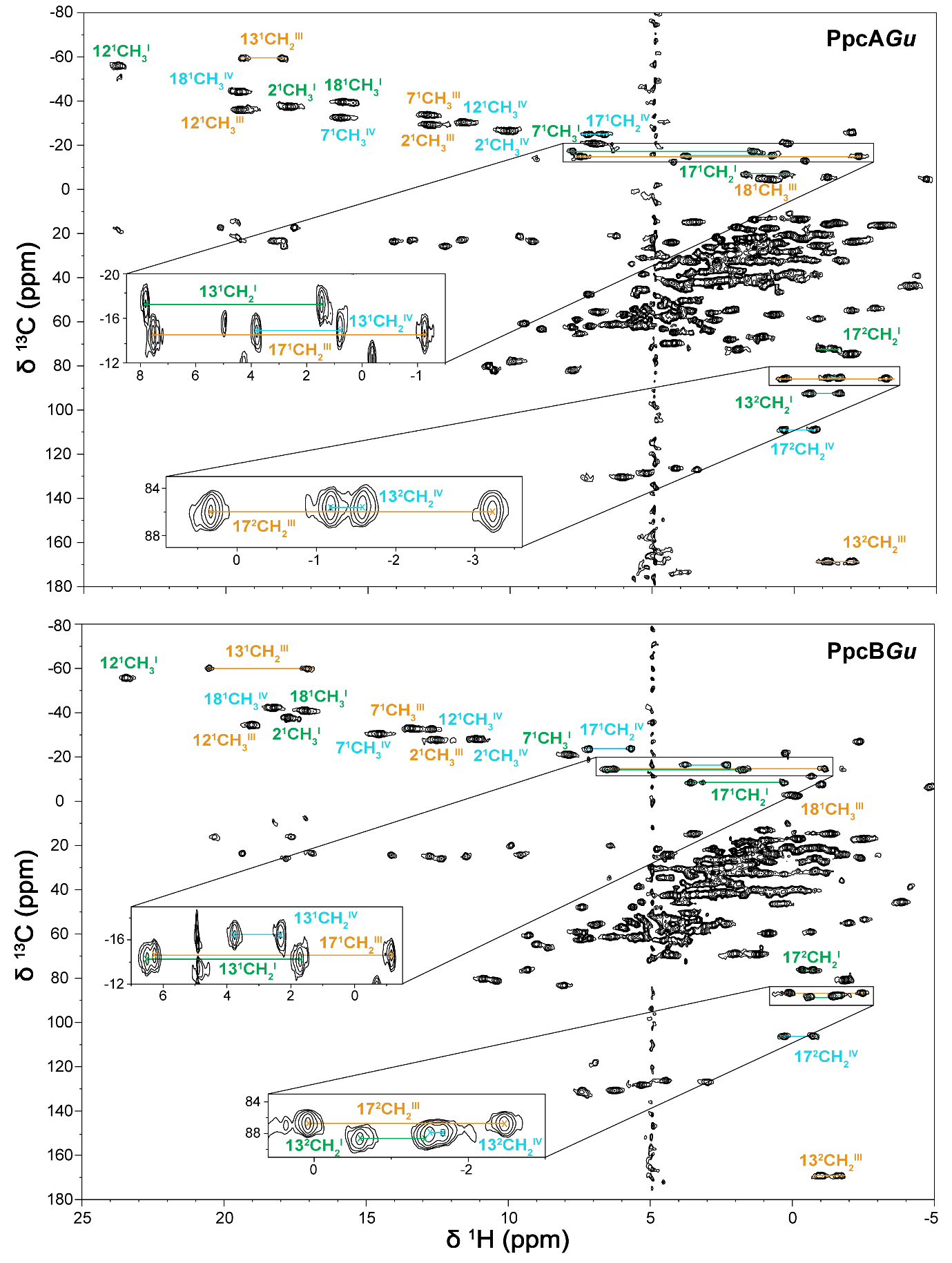


**Figure S5 – 2D ^1^H,^13^C-HMQC spectra of PpcA*Gu* (upper panel) and PpcB*Gu* (bottom panel) in the oxidized state (pH 8 and 15 °C).** The peaks of the protons connected to the same carbon atom (CH_2_ groups) are linked by a straight line. The heme signals I, III and IV are colored in green, orange and blue, respectively. The chemical shifts of the signals are listed in Table S3.

**References**

72. Pettersen, E.F.; Goddard, T.D.; Huang, C.C.; Meng, E.C.; Couch, G.S.; Croll, T.I.; Morris, J.H.; Ferrin, T.E. UCSF ChimeraX: Structure visualization for researchers, educators, and developers. *Protein Sci* **2021**, *30*, 70-82.

76. Moss, G.P. Nomenclature of tetrapyrroles. Recommendations 1986 IUPAC-IUB Joint Commission on Biochemical Nomenclature (JCBN). *Eur J Biochem* **1988**, *178*, 277-328.
